# Genome-guided Investigation of Antibiotic Substances produced by *Allosalinactinospora lopnorensis* CA15-2^T^ from Lop Nor region, China

**DOI:** 10.1038/srep20667

**Published:** 2016-02-11

**Authors:** Chen Huang, Ross Ka-Kit Leung, Min Guo, Li Tuo, Lin Guo, Wing Wai Yew, Inchio Lou, Simon Ming Yuen Lee, Chenghang Sun

**Affiliations:** 1 State Key Laboratory of Quality Research in Chinese Medicine and Institute of Chinese Medical Sciences, University of Macau, Macao, China; 2 Stanley HoCentre for Emerging Infectious Diseases, The Chinese University of Hong Kong, Shatin, New Territories, Hong Kong SAR, China; 3 Department of Microbial Chemistry, Institute of Medicinal Biotechnology, Chinese Academy of Medical Sciences and Peking Union Medical College, Beijing 100050, China; 4 Faculty of Science and Technology, Department of Civil and Environmental Engineering, University of Macau, Macao, China; 5 School of Public Health, The University of Hong Kong, Hong Kong

## Abstract

Microbial secondary metabolites are valuable resources for novel drug discovery. In particular, actinomycetes expressed a range of antibiotics against a spectrum of bacteria. In genus level, strain *Allosalinactinospora lopnorensis* CA15-2^T^ is the first new actinomycete isolated from the Lop Nor region, China. Antimicrobial assays revealed that the strain could inhibit the growth of certain types of bacteria, including *Acinetobacter baumannii* and *Staphylococcus aureus*, highlighting its clinical significance. Here we report the 5,894,259 base pairs genome of the strain, containing 5,662 predicted genes, and 832 of them cannot be detected by sequence similarity-based methods, suggesting the new species may carry a novel gene pool. Furthermore, our genome-mining investigation reveals that *A. lopnorensis* CA15-2^T^ contains 17 gene clusters coding for known or novel secondary metabolites. Meanwhile, at least six secondary metabolites were disclosed from ethyl acetate (EA) extract of the fermentation broth of the strain by high-resolution UPLC-MS. Compared with reported clusters of other species, many new genes were found in clusters, and the physical chromosomal location and order of genes in the clusters are distinct. This study presents evidence in support of *A. lopnorensis* CA15-2^T^ as a potent natural products source for drug discovery.

Natural products represent a leading source for drug candidates, especially in the anti-bacterial field^1^,^2^. Actinomycetes, regarded as a monophyletic branch of bacteria, are fungi-like bacteria forming long filaments; most importantly, these microbes tend to produce secondary metabolites such as polyketides and non-ribosomal peptides^3^,^4^. Many of these compounds have been successfully isolated and transformed into useful drugs, antibiotics, or chemotherapeutic agents^5^,^6^. Accordingly, there are more than 22,000 known microbial secondary metabolites, 70% of which are produced by actinomycetes, 20% from fungi, 7% from *Bacillus spp*. and 1–2% by other bacteria^7^.

There has been a large push towards research on the anti-bacterial mechanisms of these microbes, which can be traced to the discovery of streptomycin. Presently, researchers have been especially keen to explore new drugs by improving or activating the metabolites of microorganisms. Olano, *et al*.^8^ applied genetic approaches to bioactive secondary metabolites producing actinomycetes to improve the activity of relevant enzymes. Ito, T^8^ modified this method to improve efficacy and reduce the toxicity of actinomycete metabolites by creating new analogs using mutasynthesis. In addition, Yassin and Mankin found 77 new targets after random mutation in the RNA of a large ribosomal subunit^9^. Another difficulty for experimental work to resolve is that culture conditions can influence the expression of gene clusters in microbes.

At present, most genes and pathways involved in synthesizing various antibiotics have been identified, for example, the DOBISCUIT database collects around one hundred diverse antibiotics biosynthetic gene clusters. Moreover, Blin, *et al*. summarized previous findings on gene clusters related to different kinds of antibiotics and secondary metabolites, and automated the identification of the gene clusters encoding secondary metabolite production^10^. These two resources and alike facilitate comparison of potential antibiotics-producing gene clusters. However, “Me-Too” drugs may not help much^11^. The unremitting emergence of antibiotics-resistance also draws attention to new antibiotics source from exotic origins, such as mangrove^12^, and marine salterns^7^. Therefore, microbes from those environments may have great potential for novel antibiotics discovery^7^,^13^,^14^,^15^. It is particularly notable that microorganisms existing under extreme environmental conditions should exhibit distinctive features and abilities, and thus should be the most likely to yield unique and powerful bioactive compounds.

Schroeckh *et al*. found that *Aspergillus nidulans*, when co-cultured with bacteria, could produce several novel metabolites^16^. The capacity of secondary metabolic biosynthesis of microorganisms is always greater than that observed by fermentation^4^. Microbial genome analysis is an effective way of novel natural product producing gene discovery, due to clustering of biosynthetic genes of secondary metabolites e.g. polyketides, in genome. Beyond the known gene clusters, exploring the properties of the numerous orphan genes is regarded as the biggest challenge to finding new bioactive metabolites^17^. After genome sequencing, Udwary *et al*. found that approximately 9.9% of the marine actinomycete *Salinispora tropica* genome is related to natural product assembly, and 17 novel biosynthetic loci^4^. Hu *et al*. characterized two new cryptic sesquiterpene synthases in the *Streptomyces clavuligerus* genome by combining genetic and biochemical techniques^18^ while Soror *et al*. characterized an unusual member of the hormone-sensitive lipase family of esterases in *Streptomyces coelicolor* A3 (2)^19^. Lautru *et al*. found that in *Streptomyces coelicolor* M145 several gene clusters encoding new non-ribosomal peptide synthetase(NRPS) systems are not associated with known secondary metabolites; furthermore, they isolated and determined the structure of a new tris-hydroxamate tetrapeptide iron chelator coelichelin using a genome mining approach and guided by substrate predictions^20^.

To the best of our knowledge, strain *Allosalinactinospora lopnorensis* CA15-2^T^ is the first new actinomycete in genus level found in the Lop Nor region of the Xinjiang province of China, which is famous for its high temperature, salinity and drought^21^. Antimicrobial assays revealed that the strain could inhibit the growth of certain types of bacteria, including *Acinetobacter baumannii* and *Staphylococcus aureus*, thus, using a genomics approach to unveil and explore the potential abilities of the type strain of the new species and new genus shall guide subsequent experiments for the discovery of novel natural bioactive compounds for helping human tackle bacterial infections.

## Results

### Culture of strain CA15-2^T^ and its Antimicrobial Assay

**Figure 1:**
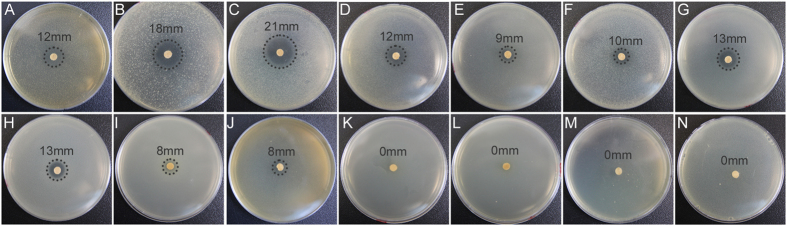
Comparison of inhibition zone diameters produced by disks among fourteen bacteria or fungi. (**A**) *Candida albicans* CCTCC AY93025; (**B**) *Acinetobacter baumannii* 2799; (**C**) *Acinetobacter baumannii* ATCC 19606; (**D**) *Escherichia coli* ATCC 25922; (**E**) *Escherichia coli* ATCC 2800; (**F**) *Klebsiella pneumonia* ATCC 700603; (**G**) *Klebsiella pneumonia* ATCC 10031; (**H**) *Staphylococcus aureus* ATCC 25923; (**I**) *Staphylococcus aureus* 2641; (**J**) *Cryptococcus laurentii* CCTCC AY91013; (**K**) *Enterococcus faecalis* ATCC 29212; (**L**) *Enterococcus faecalis* ATCC 33186; (**M**) *Pseudomonas aeruginosa* 2774; N: *Pseudomonas aeruginosa* ATCC 27853.

Better growth of strain *A.lopnorensis* CA15-2^T^ on R2A with 5% NaCl than without NaCl were observed at 28 °C pH 7.5 for 10 days (Figure S1) Antimicrobial assay shows that concentrated sample of ethyl acetate extract from the fermentation broth of strain *A*. *lopnorensis* CA15-2^T^ exhibits different degrees of inhibitory activity against two fungi and eight bacteria, in particular *Acinetobacter baumannii* 2799 and *Acinetobacter baumannii* ATCC19606 (Fig. 1).

### Genome assembly and annotation

Deep sequencing based on enzymatic digestion DNA fragmentation, yielded 1,945,282 raw reads (average read length =137 bp). After removing short, low quality and suspected-plasmid reads, 260 contigs of more than 5.8 Mb were obtained. The average coverage was 45.1 fold and the G + C content was approximately 69.61%, consistent with the result (69.60%) obtained by reverse-phase HPLC^22^. Another round of sequencing with DNA fragmentation by sonication generated 3,317,091 raw reads (average read length =222 bp), which were subsequently assembled into 1,456 contigs. In order to obtain a better assembly result, we combined the sequencing reads of the two different DNA fragmentation methods, resulting in a total of 5,200,564 reads, or 1,002,084,924 base-pairs (average read length 179.43 bp). The combined reads were assembled into 233 contigs of 5,897,123 bp, and an average G + C content of 69.61% (Table 1). The raw data and the total shotgun assembly (TSA) were submitted and archived in the GenBank under the accession number LAJC00000000 and SRS881470, under the BioProject nr PRJNA278354 and biosample nr SAMN03418058. Detailed information on sequencing and assembly is shown in Table 1. Specifically, more than 5,000 ORFs were predicted, with 5,549 protein-coding genes subjected to further annotation analysis (Figure S2). A total of 4,717 putative protein-coding genes had homologs identified in the *nr* database, with 2,987 sequences assigned to 22 functional categories by egg NOG classifications (Figure S3, A–V). The majority of these protein sequences were found to involve, in general function terms, energy and transcription. Fifty-seven RNA coding sequences were detected, including 5S, 16S and 23S rRNAs and the remaining consists of tRNAs (Table 2). The functions of remaining 832 genes cannot be detected by similarity-based search methods, suggesting that *A. lopnorensis* CA15-2^T^ may carry a novel gene pool.

### Phylogenetic and comparative analysis

**Figure 2:**
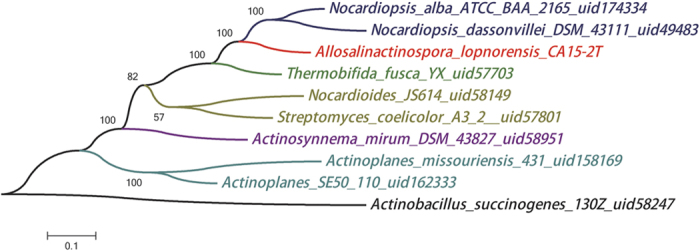
Comparison of ten housekeeping genes from strain *A. lopnorensis* CA15-2^T^ with other orthologous. Phylogenetic tree based on maximum likelihood method of a muti-alignment of ten housekeeping genes, respectively. The number of boostrap replication was set to 500.

**Figure 3:**
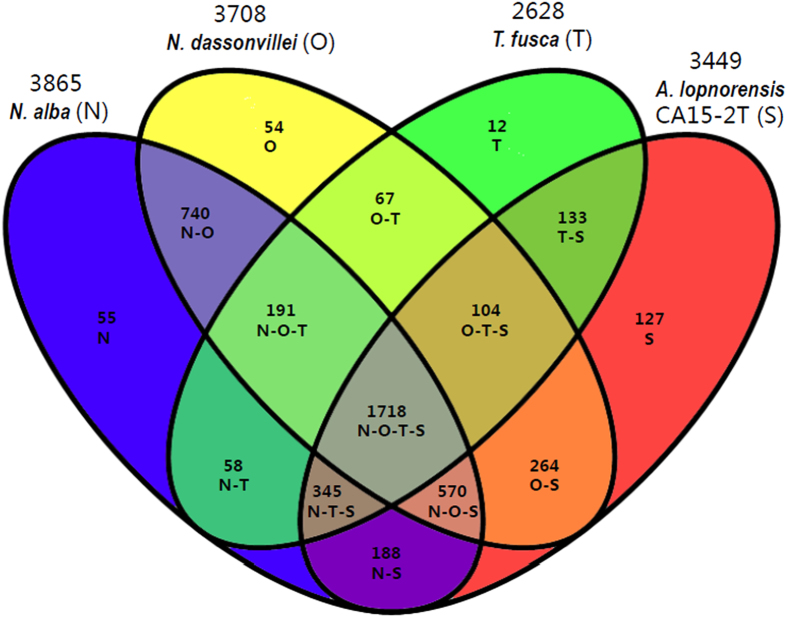
Venn diagram displaying the distribution of shared gene families among *N. alab*, *N. dassonvillei*, *T. fusca* and *A. lopnorensis* CA15-2^T^. Numbers indicate the number of gene families in each cluster. The Venn diagram was created with R software.

**Figure 4:**
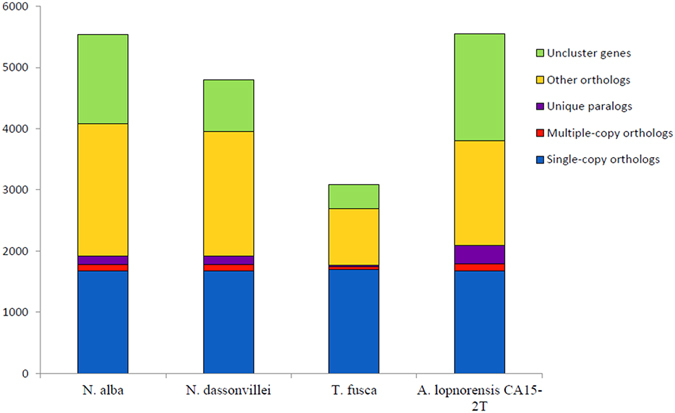
Cumulative histogram displaying the proportion of different types orthologs genes among *N. alab*, *N. dassonvillei*, *T. fusca* and *A. lopnorensis* CA15-2^T^.

Phylogenetic results based on 16S rRNA showed that *A. lopnorensis* CA15-2^T^, together with *Nocardiopsis alba* ATCC BAA2165, *Nocardiopsis dassonvillei* DSM43111, and *Thermobifida fusca* YX, are located at the same branch of the tree (Figure S4), which suggests that these species should have a close phylogenetic relationship to other species on the tree. The phylogeny constructed by house-keeping genes is in accordance with the 16S rRNA gene sequences (Fig. 2). Orthology analysis showed that 4,378 ortholog families are shared between at least two of the genomes from *N. alba* ATCCBAA2165, *N. dassonvillei* DSM43111, *T. fusca*YX or *A. lopnorensis* CA15-2^T^; 1,718 (27.6%) orthologs were shared by all four species (Fig. 3). Moreover, there were 570 (13%) families shared among all of the *Nocardiopsis* species, 127 (2.7%) families that only belonged to the strain *A. lopnorensis* CA15-2^T^. For those genes involved in the orthologous family, all four species had similar numbers (approximately 1,600) of single-copy orthologous genes (Fig. 4). In addition, 311 unique paralogous genes, belonging to 127 unique families, were found in *A. lopnorensis* CA15-2^T^, by contrast, *N. dassonvillei* has 128 unique paralogous genes, *N. alba* has 138 ones and *T. fusca* has only 26 unique paralogous genes, The larger amount of unique paralogous A. l*opnorensis* CA15-2^T^ might associated with its unequalled features of adaptation to extreme environments and capacity of potential novel secondary metabolites biosynthesis.

Four genes encoding the enzymes for ectoine (1,4,5,6,tetra-2-methyl-4-pyrimidonecarboxylic acid) synthesis were identified. The four putative ectoine biosynthetic genes are located very close one after the other (Table 3) and the order of these four genes is highly similar to those of other halophiles of actinomycete, e.g. *Streptomyces coelicolor* A3 (2), *Nocardia farcinica* IFM10152 and *T. fusca* YX^23^, which provides evidence for the molecular basis of adaptation to a saline environment (Figure S1).

### Antibiotics-producing Capability Assessment

Our antimicrobial assay revealed that *A. lopnorensis* CA15-2^T^ inhibit the growth of specific bacteria, and gene cluster prediction also indicated that it may contain novel gene clusters for antibiotics synthesis. Here, a comparative analysis of *A. lopnorensis* CA15-2^T^ with 10 diverse antibiotic-producing bacteria, as well as 10 non-antibiotic-producing bacteria was conducted to assess the capacity of producing antibiotics, and a new measurement Q was proposed, in which not only matched similarity (identity value) and length (alignment length) were taken into account, but also the gene amount was used for normalization, in terms of the higher the gene number, the greater the probability of gene sets of this species matching the sequences of gene clusters involved in antibiotic production. The results indicated that the majority of Q values of the antibiotic-producing group were markedly larger than those of non-antibiotics-producing group (Tables 4 and 5). It should be noted that some gene clusters of biosynthetic antibiotics (e.g. Avermectin, Myxothiazol) showed no obvious difference between the antibiotics-producing and non-antibiotic-producing groups merely based on a count of the hit number of gene/PKS genes during the blast search (Table S1,S2), whereas exhibited obvious differences in Q values (Tables 4 and 5). Furthermore, statistical test repeated measures ANOVA for the comparison of antibiotics-producing and non-antibiotic-producing groups showed that the difference in Q value between the antibiotics-producing and non-antibiotic-producing groups is statistically significant. Concretely speaking, multivariate test indicated that the selection of gene cluster did not affect the efficiency of Q-value (P value = 0.001, Table S3), and test of Between-Subjects Effects showed that Q value between the antibiotics-producing and non-antibiotic-producing groups is significantly different (P value ≤0.05, Table S4). On the other hand, twenty one bacteria including ten antibiotics-producing bacteria and ten non-antibiotic-producing bacteria as well as *A. lopnorensis* CA15-2^T^ was classified using principal component analysis (PCA) on Q value, which illustrated that *A. lopnorensis* CA15-2^T^ tend to cluster with antibiotics-producing group (Figure S5). Similarly, cluster and phylogenetic analysis of Q value revealed that the strain *A. lopnorensis* CA15-2^T^, and the antibiotics-producing species, were placed in the same group (Figure S6,S7), pointing toward the potential presence of the antibiotics-producing capacity in the strain *A. lopnorensis* CA15-2^T^.

### Detection of gene clusters involved in Antibiotics & Secondary Metabolites

**Figure 5:**
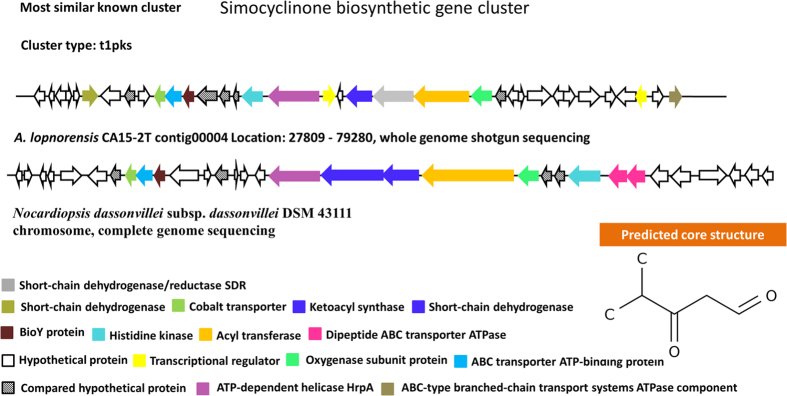
Proposed biosynthetic gene cluster of simocyclinone-like compound in *A. lopnorensis* CA15-2^T^. The most similar gene cluster from *Nocardiopsis dassonvillei* is shown, with related genes depicted in the same color to highlight intracluster gene rearrangements. Predicted core structure displayed in the lower right corner.

**Figure 6:**
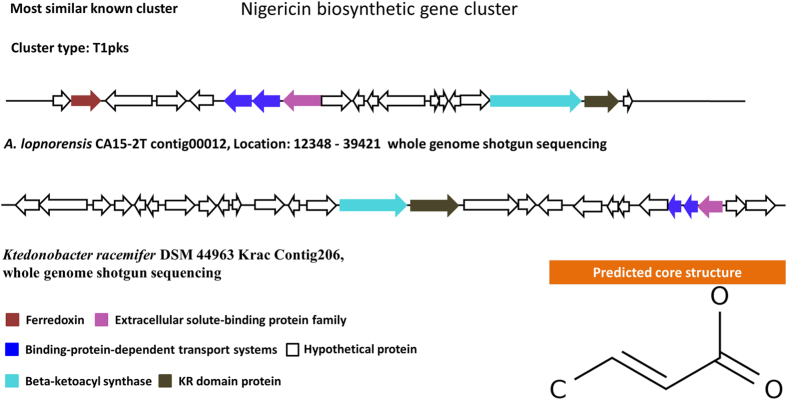
Proposed biosynthetic gene cluster of nigericin-like compound in *A. lopnorensis* CA15-2^T^. The most similar gene cluster from *Ktedonobacter racemifer* is shown, with related genes depicted in the same color to highlight intracluster gene rearrangements. Predicted core structure displayed in the lower right corner.

**Figure 7:**
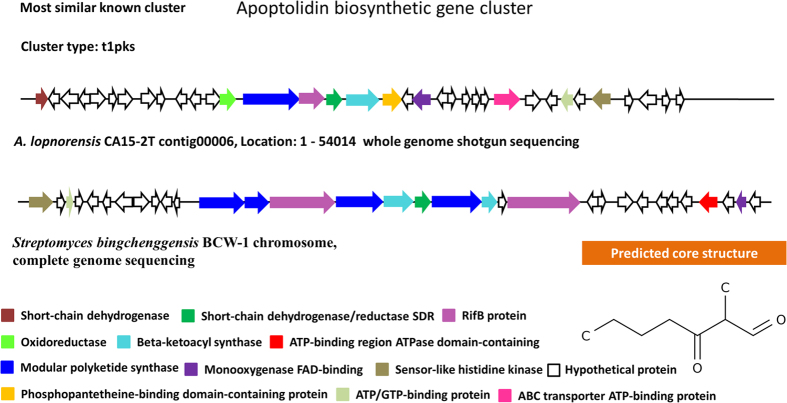
Proposed biosynthetic gene cluster of apoptolidin-like compound in *A. lopnorensis* CA15-2^T^. The most similar gene cluster from *Streptomyces bingchenggensis* is shown, with related genes depicted in the same color to highlight intracluster gene rearrangements. Predicted core structure displayed in the lower right corner.

**Figure 8:**
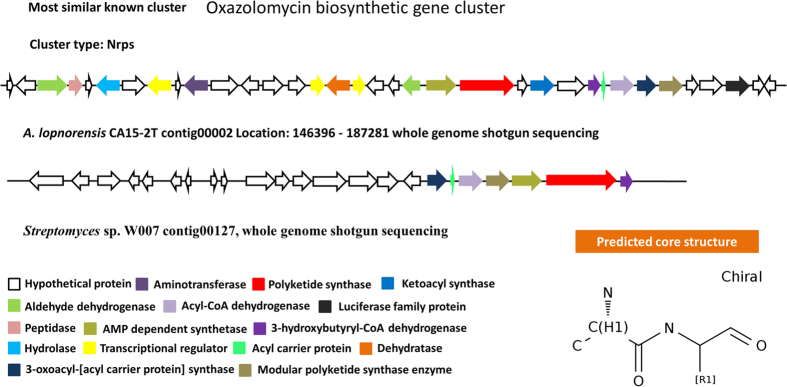
Proposed biosynthetic gene cluster of oxazolomycin -like compound in *A. lopnorensis* CA15-2^T^. The most similar gene cluster from *Streptomyces* sp. W007 is shown, with related genes depicted in the same color to highlight intracluster gene rearrangements. Predicted core structure displayed in the lower right corner.

A total of 17 secondary metabolic biosynthesis gene clusters were identified in *A. lopnorensis* CA15-2^T^, which are predicted for polyketide, non-ribosomal peptide, siderophore, terpenoid and other products (Table 6). The combined length of these gene clusters was estimated at 412 kb. Specifically, the analysis shows that the genome of *A. lopnorensis* CA15-2^T^ may contain at least six kinds of type I PKS gene clusters (Table 6), in which three core structures were identified using anti SMASH prediction. In the present study, the analysis revealed that four of the above PKS I gene clusters are likely to be involved in the biosynthesis of simocyclinone, apoptolidin, nigericin and oxazolomycin. The simocyclinone biosynthesis gene cluster of *A. lopnorensis* CA15-2^T^ contains 35 ORFs (Fig. 5), five of which encode modular PKS. These modular PKSs contain 13 catalytic domains in which acyl-chain elongation involves acyl carrier protein (ACP), β-ketoacyl-ACP reductase (KR), β-ketoacyl-ACP synthase (KS), acyltransferase (AT) and dehydratase (DH). In addition, the simocyclinone cluster of *A. lopnorensis* CA15-2^T^ is relatively similar to the existing simocyclinone cluster of *Nocardiopsis dassonvillei* subsp. *dassonvillei* DSM 43111 in aspects of amino acid sequence (Figure S8,S9), ORF order as well as composition, particularly with respect to several homologous putative proteins (white-striped arrows in Fig. 5) that may play a role in the synthesis of the simocyclinone-derived primer unit, which indicates that these clusters may be derived from donor microorganisms via horizontal gene transfer. Another type I PKS gene cluster may produce nigericin-like compound with high similarity to *Ktedonobacter racemifer* DSM 44963. The type I PKS cluster contains 19 ORFs, of which 5 encode modular PKS. Likewise, both clusters are composed of numerous hypothetical unknown function proteins (Fig. 6), and the majority of the key PKS genes share high amino acid similarity (Figure S10). Furthermore, an apoptolidin-like PKS I biosynthetic gene cluster was found in the *A. lopnorensis* CA15-2^T^ genome. Comparison of both clusters indicates that apoptolidin-like cluster has several novel members that are not present in the *Streptomyces bingchenggensis* BCW-1 cluster (Fig. 7), including oxidoreductase, short-chain dehydrogenase and phosphopantetheine-binding domain-containing protein. However, the apoptolidin cluster of *Streptomyces bingchenggensis* BCW-1 has more polyketide synthase than the *A. lopnorensis* CA15-2^T^ cluster. In addition to the abundance of the diverse PKS cluster, *A. lopnorensis* CA15-2^T^ harbors a non-ribosomal peptide synthetase (NRPS) pathway. The NRPS cluster is composed of 36 ORFs, of which 8 encode key PKS modular with 13 domains. Compared with the most-similar known cluster of *Streptomyces* sp. W007 contig 00127, the cluster of *A. lopnorensis* CA15-2^T^ has several unique ORFs including Hydrolase, Ketoacyl synthase and transcriptional regulators, which indicates that *A. lopnorensis* CA15-2^T^ may have the potential of synthesizing an oxazolomycin-analog (Fig. 8).

### Secondary metabolites Identified Directly by Mass Spectrometric Analysis

**Figure 9:**
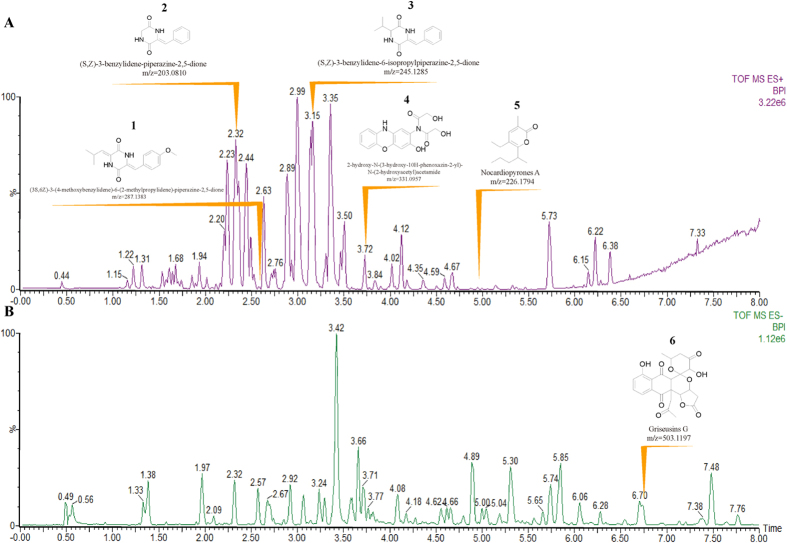
High-resolution LC-MS analysis of ethyl acetate (EA) extract of the fermentation broth of strain *A.lopnorensis* CA15-2^T^. The chromatographic separation was performed on an ACQUITY UPLC HSS T3 column (2.1 × 100 mm). Detailed characterization of separated metabolites was executed with a QTof mass spectrometer outfitted with a high transmission efficiency collision cell (Xevo G2-XS QTof, Waters, Milford, MA). Two ESI ionization modes in MS analysis, positive (**A**) and negative (**B**), were carried out respectively to give the diverse molecular properties in the EA extract. Structures and mass-to-charge ratios* of the six antibiotics or antibiotic substances (compounds 1–6) show upon their specific retention times correspondingly. *For the six antibiotics or antibiotic substances: compounds 1–5 in Positive ionization mode (Nocardiopyrones A: [M + NH4]+; others: [M + H]+); compound 6 in Negative ionization mode (Griseusins G: [M-HCOO]−).

Mass spectra of the ethyl acetate (EA) extract of the fermentation broth of the strain CA15-2^T^ generally exhibited a large number of discrete ions ranging from m/z 50 to 1200 both in positive and in negative ionisation modes. From UPLC-MS profiles, a total of 92 metabolic substances were identified by UNIFI workstation according to the mass-to-charge ratio of molecular ions and their corresponding fragment ions. Amongst them, six compounds, possessing antibacterial, antifungal or antitumor activity, were concerned (Fig. 9: compound 1–6). They affiliate to four groups: diketopiperazines (DKPs) group^24^ (compound 1–3), phenoxazine derivatives^25^ (compound 4), alpha-pyrone group^26^ (compound 5), and pyranonaphthoquinone (PNQ) group^27^ (compound 6). Concretely speaking, three diketopiperazines (DKPs) compounds (1–3) produced by a deep-sea-derived *Nocardiopsis alba* SCSIO 03039 formerly reported did not showed any antibacterial activities against four indicator strains *Escherichia coli* ATCC 25922, *Staphylococcus aureus* ATCC 29213, *Bacillus subtilis* SCSIO BS01 and *Bacillus thuringiensis* SCSIO BT01^24^. The antibacterial efficacy of Phenoxazine derivative (4) produced by a marine derived strain, *Nocardiopsis* sp. 236 against *M. smegmatis* was tested, yet it didn’t show any antibacterial activity^28^. Nocardiopyrones A (5) produced by *Nocardiopsis alkaliphila*, an alkalophilic actinomycete showed weak antibacterial activities against *Pseudomonas aeruginosa*, *Enterobacter aerogenes* and *Escherichia coli* with MIC values of 20, 40 and 40 μM and showed no activity against both *Staphylococcus aureus* and *Candida albicans* with more than 100 μM of MIC values^26^. Griseusins G (6) produced by *Nocardiopsis* sp. YIM DT266, also an alkalophilic actinomycete, showed strong inhibitory activity against *Staphylococcus aureus* ATCC 29213, *Micrococcus luteus*, and *Bacillus subtilis* with MIC values of 0.8, 1.47 and 1.33 μg/ml, respectively^29^. Antimicrobial results of the six known compounds identified by UPLC-MS revealed that only Griseusins G contributed inhibitory activity against Gram-positive bacteria, such as *Staphylococcus aureus*, but none showed any activities against Gram-negative bacteria, such as *Escherichia coli*, *Klebsiella pneumonia*, especially *Acinetobacter baumannii*, against which the biggest transparent inhibition zone was produced by the strain *A. lopnorensis* CA15-2^T^ (Fig. 1B,C). The marked conflict between MS elucidation and antimicrobial assay implies the existence of novel antibiotic substance.

## Materials and Methods

### Strains and Growth Media

Strain *A. lopnorensis* CA15-2^T^ was deposited in Institute of Medicinal Biotechnology, Chinese Academy of Medical Sciences & Peking Union Medical College and maintained on R2A (Difco, BD, USA) slants containing 5% (w/v) NaCl at 4 °C or as a 20% (v/v) glycerol suspension at −20 °C. Growth features of strain *A. lopnorensis* CA15-2^T^ were evaluated on R2A medium with 5% NaCl and without NaCl at 28 °C, pH 7.5 for 10 days. Both the seed medium and fermentation broth was tryptic soy broth (TSB; Bacto^TM^, BD, USA) supplemented with 5% (w/v) NaCl. Two fungi strain, *Candida albicans* CCTCC AY93025 and *Cryptococcus laurentii* CCTCC AY91013 were obtained from China Center for Type Culture Collection in Wuhan City, Hubei Province of China and were propagated in Sabouraud Dextrose Agar (SDA, Oxoid, UK) or Sabouraud Dextrose Broth (SDB, Oxoid, UK). Eight standard strains including *Acinetobacter baumannii* ATCC 19606, *Escherichia coli* ATCC 25922, *Klebsiella pneumonia* ATCC 10031 and ATCC 700603, *Staphylococcus aureus* ATCC 25923, *Enterococcus faecalis* ATCC 33186 and ATCC 29212, *Pseudomonas aeruginosa* ATCC 27853 were obtained from American Type Culture Collection (ATCC, Rockville, MD). Four drug-resistant strains: *Acinetobacter baumannii* 2799, *Escherichia coli* 2800, *Staphylococcus aureus* 2641 and *Pseudomonas aeruginosa* 2774 were obtained from Beijing Hospitals. Mueller-Hinton broth (MHB; Oxoid, UK) was used as the cultivation medium of all 12 bacteria strains.

### Antimicrobial Assay

Antimicrobial assay were carried out by paper-disc agar diffusion method^30^. The stock culture of strain CA15-2^T^ was inoculated into 250 mL Erlenmeyer flasks containing 50 mL of TSB (BD) supplemented with 5% (w/v) NaCl as seed culture medium, the seed culture was harvested after 5 days incubation at 37 °C on a rotary shaker at 180 rpm and then 50 mL of culture broth were transferred to 1 L of producing medium with the same ingredients as seed culture medium in 5 L Erlenmeyer flasks. Four sets of 5 L Erlenmeyer flasks were incubated in the same culture condition as the seed culture. Four liters of the fermentation broth were harvested after incubation for 10 days and further centrifuged at 8000 rpm for 10 min at room temperature. Four liters of supernatant were extracted with the same volume of ethyl acetate and the organic layer was further concentrated to dryness under reduced pressure, and then finally was dissolved in 2 mL methanol as sample for test. Forty microlitres sample were loaded on sterilized filter paper with a diameter of 6 mm. After dryness, filter papers were covered on the agar plate containing different fungi or bacteria as indicator strain and antimicrobial activities were observed and recorded after 24 hours incubation at 37 °C and the zones of inhibition were measured.

### Mass Spectrometric Analysis

Concentrated sample of EA extracts was analyzed on ACQUITY^TM^ Ultra Performance Liquid Chromatography system (Waters, USA). The chromatographic separation was performed on a ACQUITY UPLC HSS T3 column (2.1 × 100 mm I.D., 1.8 μm). The mobile phase consisted of water containing 0.1% formic acid (A) and acetonitrile (B). The gradient condition was 1–99% B at 0–10 min, 99% B at 10–11 min, 99-1% B at 11–13 min. The flow rate was 0.45 mL/min.

Detailed characterization of each peaks separated by UPLC was performed with a Xevo^TM^ G2-XS QTof (Waters, Manchester, UK), quadrupole and orthogonal acceleration time-of-flight tandem mass spectrometer. In negative ion mode capillary voltage was set to 2.5 kV, source temperature to 120 °C, desolvation temperature to 500 °C, and sample cone voltage to 40 V. In positive ion mode capillary voltage was set to 3.0 kV, source temperature to 120 °C, desolvation temperature to 500 °C, and sample cone voltage to 40 V. In both modes, mass spectra were acquired at a speed of 0.2 s/scan and the scanning delay of 0.01 s during analysis. In the MS^E^ experiments, collision energy was set to scan between 6 eV and 15–45 eV. The analysis results were acquired with the lockspray to guarantee accuracy Leucine-enkephalin was used as the lockmass at a concentration of 400 ng/mL and flow rate of 10 μL/min. Data acquisition and analysis were controlled by Waters UNIFI V1.71 software. The scan rang in MS and MS/MS modes were over a range of 50–1200 m/z.

### Genome Sequencing, Assembly and Annotation

The genomic DNA library was constructed using the Ion Xpress Plus Fragment Library Kit (Life Technologies) after enzymatic digestion. The library was sequenced using the Ion Torrent platform. Since DNA fragmentation bias can lead to uneven coverage, we constructed another library with the sonication fragmentation method that was also sequenced using the Ion Torrent platform. Genome assembly of the pooled sequencing reads was performed by the Newbler program, after removing short, low-quality reads. Glimmer (ver. 3.0)^31^ and Prodigal^32^ were used for gene prediction. The latter was used to minimize prediction errors by Glimmer for high GC prokaryotes^33^. The prediction results of longer gene length were adopted if the location sites of genes overlap by the two prediction methods. Transfer RNAs (tRNAs) were predicted by tRNAscan^34^, and ribosomal RNAs (rRNAs) by RNAmmer^35^. All the putative protein-coding genes were searched against the non-redundant protein sequence (NR) database of the National Center for Biotechnology Information (NCBI) using BLASTP^36^ for annotation. The functional category was assigned by searching against eggNOG 3.0^37^.

### Phylogeny and Genome Comparison

To demonstrate a phylogenetic evolutionary relationship with other species, the complete 16S rRNA gene sequence of *A. lopnorensis* CA15-2^T^ was retrieved from the annotation results. Lin Guo *et al*. have demonstrated that this species is a new member of family *Nocardiopsaceae* based on phylogenetic analysis using 16S rRNA sequence^22^. In this study, we added nine affinitive species of *A. lopnorensis* CA15-2^T^ which had been deposited in the NCBI database to further verify the branches. These species are *Nocardiopsis alba* ATCC BAA 2165 (Biosample accession: SAMN02603688)*, Nocardiopsis dassonvillei* DSM43111 (Biosample accession: SAMN02598425), *Thermobifida fusca*YX (Biosample accession: SAMN02598543), *Nocardioides sp*. JS614 uid58149 (Biosample accession: SAMN02598280)*, Streptomyces coelicolor* A3 (2) uid57801 (Biosample accession: SAMEA1705940)*, Actinobacillus succinogenes* 130Z uid58247 (Biosample accession: SAMN02598296)*, Actinoplanesmissouriensis*431 uid158169*, Actinoplanes sp*. SE50 110 uid162333 (Biosample accession: SAMN02603081) and *Actinosynnemamirum*DSM 43827 uid58951 (Biosample accession: SAMN00001904). Sequence alignment was done using MUSCLE^38^. The aligned 16S rRNA gene sequences were used to reconstruct the phylogeny using Maximum Likelihood (ML) algorithm by MEGA^39^. For cases of multiple 16S rRNA gene copies in the genome, the longest sequence was chosen for phylogenetic analysis. In addition, 10 house-keeping genes (acsA, aroE, dnaE, guaA, gyrB, mutL, ppsA, pyrC, recA and rpoB)^40^ were concatenated to reconstruct the phylogeny for comparison with the one by 16S rRNA. Three species with *A. lopnorensis* CA15-2^T^ in the same branch of the phylogenetic tree (Fig. 2) were used for further comparative analysis by the Ortho MCL software package^41^, which was used to identify ortholog, inparalog and co-ortholog groups. Finally, overlapping of the ortholgous families were visualized in a Venn diagram created by the R software package.

### Identification and assessment of antibiotics-producing of gene clusters involved in secondary metabolites

The assembled contigs of *A. lopnorensis* CA15-2^T^ were submitted to the anti SMASH2.0 server to search for potential secondary metabolic biosynthetic gene clusters^10^. Type I pks gene clusters, in which core structures were identified in anti SMASH detection were extracted for comparison with known gene clusters of other species using BLAST^36^. Multiple amino acid alignment of core proteins was performed by ClustalW^42^ and depicted by boxshade (http://www.ch.embnet.org/software/BOX_form.html). On the other hand, in order to assess the antibiotics-producing capacity of *A. lopnorensis* CA15-2^T^, 15 gene clusters involved in synthesizing the known diverse antibiotics were randomly extracted from the DOBISCUIT database^43^ (Table S5) to be compared with the genes of *A. lopnorensis* CA15-2^T^ using BLAST, respectively. Furthermore, 10 gene sets from 10 known antibiotic-producing bacteria, which were picked up according to literature retrieval^44^,^45^,^46^,^47^,^48^,^49^,^50^ (Table S6), were also compared with the 15 gene clusters, respectively. Ten non-antibiotics-producing bacteria were retrieved from the NCBI database as the control group (Table S6), and the corresponding gene sets of these bacteria were also compared with the 15 gene clusters, respectively. It should be noted that none of research investigated which bacteria could not produce antibiotics, in such case; we select ‘specific’ bacteria as non-antibiotics-producing bacteria which might not to be expected to produce antibiotics, e.g. pathogenic bacteria, acid-tolerant, halo tolerant or heat-resistant bacteria. In order to evaluate the antibiotics-producing capacity of these ten antibiotic-producing bacteria and ten non-antibiotics-producing bacteria, as well as *A. lopnorensis* CA15-2^T^, index Q value was developed, in which the identity and alignment length were taken into account, and gene amount was used for normalization, defined as:


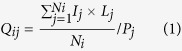


where **I**_**j**_ is the best-matched identity value to the blast result (**j**th species compared with the **i**th gene cluster), **L** is the best-matched alignment length of the blast result, and **N**_i_ is the gene number of the **i**th gene cluster and **P**_**j**_ is the gene amount of the **j**th species. The larger **Q**, the greater probability of the **j**th species contains the **i**th gene cluster. Moreover, Repeated Measures ANOVA test and Principal Component Analysis was used to test the significance of difference between antibiotics-producing group and non-antibiotics-producing group.

## Conclusion

Combining genome-scale physiologic, genetic, and metabolic investigation approaches will expand our knowledge pertaining to the exploitation of novel natural active compounds, and may help us to establish strategies to develop these natural products for human use. In this study, we sequenced the genome of a novel actinobacteria strain, *A. lopnorensis* isolated from the rhizophere of Tamarisk in the Lop Nor region, an extreme environment with hot, drought, and high salinity conditions. From the secondary metabolites predicted with the assembled genome, we identified 17 candidate gene clusters, of which 4 are novel; the remaining 13 clusters refer to type I polyketide synthase (PKS I) systems, nonribosomal peptide synthetases (NRPS) and ectoine. Regarding the known gene clusters, the majority of the genes, and several new members, were found in clusters of the 13 detectable secondary metabolites. Some genes’ loci and directions changed significantly compared to those in reported clusters, and some new members joined these clusters. The findings reflect the flexibility and evolutionary diversity of clusters of secondary metabolites, and also bring new insights for future research on the secondary metabolites of actinomycetes.

## Discussion

Bacteria are rich resources for the production of secondary metabolites, which represent a great diversity of economically important chemicals. Actinomycetes, one of the most-important bacterial species, have been, and continue to be, a leading source for new drug discovery^1^. In recent years, the majority of actinomycetes-based screening programs at large pharmaceutical companies encountered hurdles due to high costs and frequent re-discovery of known compounds, leading to the redundancy^6^. The discovery of new strains, from extreme environments, has become a hot topic because it provides opportunities for the discovery of novel chemical structures and scaffolds with new biological functions. In the present study, *A. lopnorensis* CA15-2^T^ was isolated from the Lop Nor region. Lop Nor, which translates to “Lop Lake” in Mongolian, was a former salt lake, and is now completely dried-up. Few forms of life, except some special microbial populations, can exist in such extreme environment^51^,^52^. Culture experiment shows that *A. lopnorensis* CA15-2^T^ grew well in the medium with 5% NaCl. These microorganisms have evolved into halophilic bacteria and archaea with the development of hypersaline habitats, e.g. evaporation of marine saltwater. Generally speaking, microorganisms of this group may accumulate highly water-soluble organic compounds, which are often named ‘compatible solutes’^53^, to maintain osmotic equilibrium with surrounding medium; thus, they can keep their cytoplasm and the cell’s interior remains basically unchanged. The majority of compatible solutes are the amino acid derivatives glycine-betaine and ectoine in halophilic bacteria^52^. In this study, ectoine biosynthetic gene cluster was detected using whole genome sequencing. The metabolic pathway of Ectoine had been worked out by previous studies^54^,^55^. Briefly, aspartate-semialdehyde is transaminated to 2,4-diaminobutyric acid (DABA) by catalyzing with DABA transaminase (EctB), and DABA is then transferred to Nγ-acetyl-L-2,4-diaminobutyric by DABA-Nγ-acetyltransferase (EctA). Finally, ectoine synthase (EctC) catalyzes the cyclic condensation of Ng-acetyl-L-2, 4-diaminobutyric acid, which leads to the formation of ectoine. Ectoine hydroxylase (EctD) can convert some of the ectoine to 5-hydroxyectoine under certain stress conditions (Figure S11). The identification of Ect ABCD genes based on deep sequencing facilitate us to understand the adaptation of *A. lopnorensis* CA15-2^T^ to a salty environment.

Concerning antimicrobial experiments, *A. lopnorensis* CA15-2^T^ showed a wide-ranging antimicrobial activity, in particular effectively inhibiting the growth of *Acinetobacter baumannii*, which provides clues to the possible presence of secondary metabolites as antibacterial substances. *Acinetobacter baumannii* belonging to Nonfermentative Gram-negative bacilli, has aroused wide concern in clinical and microbiology research, due to the spread of multidrug-resistant (MDR) bacillary strains among critically ill, hospitalized patients, sometimes in form of epidemics^56^,^57^. Multidrug-resistant *Acinetobacter baumannii* has been recognized to be among the most difficult to treat gram-negative bacilli^58^. Hence, discovery of novel antibiotics in *A. lopnorensis* CA15-2^T^ undoubtedly brings new hope for fighting against *Acinetobacter baumannii*. Mass spectrometric analysis reveals 6 compounds with antifungal or antitumor activity. However, only Griseusins G among these compounds demonstrated inhibitory activity against Gram-positive bacteria (i.e., *Staphylococcus aureus*). None of them showed any activities against Gram-negative bacteria (i.e., *Acinetobacter baumannii*). In contrast to the antibiotic action merely against Gram-positive bacteria by known compound, findings from antimicrobial assay may infer a novel, yet antibiotic-related gene pool, and compounds encoded by them, in *Allosalinactinospora lopnorensis* CA15-2^T^. Normally, secondary metabolites of a microorganism are catalyzed by a series of enzyme-encoding genes that are usually organized and located in a specific section of the genome, also known as gene clusters. This feature allows us to identify potential biosynthetic clusters based on genome mining approach, e.g. basic homology sequence search BALST against existing gene clusters. To date, the genome-guided approach has been used in studies of microbial natural products^4^. Complete genome sequencing has provided unparalleled access to secondary metabolite producing genes, especially using high-throughput sequencing techniques, which have been widely used to successfully discover natural products in microorganisms for several years^1^,^4^. Furthermore, whole genome analysis in microorganisms is increasingly being performed by diverse research, clinical and public health laboratory groups^59^. In this study, the genome of *A. lopnorensis* CA15-2^T^ was sequenced using the Ion Torrent PGM platform. Two different DNA fragmentation methods were used to construct a DNA library. Although high G + C content, multicopy segments in the genome and sequencing errors might cause frequent termination of contig assembly, which is not easily filled by conventional sequencing methods, two sequences could provide sufficient sequence and complementary information for further analysis. Through biosynthetic cluster detection, 17 known or predicted gene clusters were figured out, majority of clusters were involved in production of antibiotic substances, including several well-known compounds synthetic pathways like simocyclinone, apoptolidin and oxazolomycin. Concretely speaking, simocyclinone is active against gram-positive bacteria and some studies have showed that it may process distinct cytostatic activities against human tumor cell lines^60^,^61^. In addition, Ruth H. Flatman *et al*. demonstrated that it may inhibit an early step of the gyrase catalytic cycle by preventing binding of the enzyme to DNA^62^. Apoptolidin has been proved to be a selective cytotoxic macrolide compound^63^, which can be produced by the polyketide synthesis gene cluster of *Streptomyces bingchenggensis* BCW-1. Some studies showed that apoptolidin exhibit selective activity against cells, and it could induce cell death in malignant cell lines when applied together with LDH inhibitor oxamate^64^,^65^. Furthermore, oxazolomycin that might be synthesized by NRPS cluster in *A. lopnorensis* CA15-2^T^, has been proven to show diverse and important antibacterial, antitumor and anti-human immunodeficiency virus activity^66^.

Remarkably, the genes’ loci and directions changed, and some orphan genes were found in some of these clusters, which implies *A. lopnorensis* CA15-2^T^ probably produces novel antibiotics. Given the transcriptional status and common co-existence of transcriptional regulators in gene clusters of secondary metabolites, the loci of genes might influence the final assembly of the secondary metabolic complex, because different transcription factors may regulate the expression level of genes. Likewise, the direction of genes may regulate expression in transcription levels. Moreover, metabolic substance’s gene cluster might be silenced by a specific regulation mechanism in genome^67^, and just expressed under particular conditions, which might be one of reasons that we could not identify the specific products from MS analysis. Another probably reason is that *A. lopnorensis* CA15-2^T^ has the capacity of producing other classes of antimicrobial substances, such as antimicrobial peptides, which could not be detected by MS analysis. Actually, another major class of antimicrobial agents is composed of ribosomally synthesized and post-translationally modified peptides (RiPPs), which serve as a promising alternative to conventional antibiotics biosythesized by polyketides or non-ribosomal pathways^68^,^69^. In order to test this hypothesis, genome scanning was carried out using BAGEL3 server^70^. The results revealed that *A. lopnorensis* CA15-2^T^ genome might encode two RiPPs: Linear azol(in)e-containing peptide (LAP) (Figure S12) and Lasso peptide (Figure S13). LAPs have been widely identified by mining publicly available genome sequences. According to previous studies^68^,^69^, the critical components in LAP biosynthesis are the inactive precursor peptide and the heterotrimeric synthetase complex comprised of a dehydrogenase and cyclodehydratase, all of which were identified in *A. lopnorensis* CA15-2^T^ (Figure S12). In addition, some LAPs have been proven to exhibit antibiotic activity, e.g. Goadsproin produced by Streptomyces sp. TPA0584^69^. Lasso peptides, the most extraordinary RiPPs, are regarded as promising antibacterial peptides due to their specific knotted structure^71^, the lasso fold that gives them stability against heat, chemical attack and proteases^72^. Previous studies^69^ on the biosynthesis of Lasso peptide have revealed that at least four encoding genes are essential for its formation. Three of them were detected in the genome of *A. lopnorensis* CA15-2^T^ except for the gene encoding transporter (Figure S13), which may transport the lasso peptide out of cell. One possible reason is that incomplete genome assembly of *A. lopnorensis* CA15-2^T^ leading to the interruption of gene cluster, or the gene of *A. lopnorensis* CA15-2^T^ is so distinct with the existing ones that we could not identify it based on sequence similarity search. Majority of Lasso peptides are enzyme inhibitors or receptor antagonists, more importantly, some of them have been proven to possess antibacterial activity against either Gram-negative bacteria or Gram-positive bacteria^69^. This finding may provide the clue to interpret that *A. lopnorensis* CA15-2^T^ has the capability of inhibiting against *Acinetobacter baumannii*. Certainly, further experimental investigation is requisite to validate the truth of these RiPPs in *A. lopnorensis* CA15-2^T^, e.g. proteomics approach. In conclusion, this study demonstrated that *A. lopnorensis* CA15-2^T^ has enormous potential to antibiotic development. Despite efforts and progress made towards gene function identification in the field of analytical chemistry, it’s still not easy to connect the detected genotypes with chemotypes, indicating the identification of these metabolites remains a big challenge.

## Additional Information

**How to cite this article**: Huang, C. *et al*. Genome-guided Investigation of Antibiotic Substances produced by *Allosalinactinospora lopnorensis* CA15-2^T^ from Lop Nor region, China. *Sci. Rep*. **6**, 20667; doi: 10.1038/srep20667 (2016).

## Supplementary Material

Supplementary Information

## Figures and Tables

**Table 1:** Genome sequencing and assembly statistics.

Sequence data	Enzymatic digestion			Sonication		
	Insert size (bp)	High-quality data (Mb)	Sequence depth (fold coverage)	Insert size (bp)	High-quality data (Mb)	Sequence depth (fold coverage)
Raw data	5–353	266.2	45.13	5–395	736.3	124.83
Filter data	50–353	202.9	34.45	50–395	684.9	116.28
	Data merge					
Assembly statistics	ContigN50 (bp)	61350				
	Max length (bp)	231302				
	GC content (%)	69.61				
	Total size (Mb)	5.89				

**Table 2:** General features of the genome of *A. lopnorensis*CA15-2^T^.

Contigs	233
DNA, total number bases	5894259
Coding density (%)	85.4
G + C content (%)	69.61
Genes, total number	5662
Protein coding genes	5605
Total RNA genes	57
5S rRNA	1
16S rRNA	1
23S rRNA	1
tRNA	54

**Table 3:** Detailed information of four genes encoding enzymes involved in Ectoine synthesis of *A. lopnorensis* CA15-2^T^ located in assembled sequence (contig00008).

Gene ID	Coding enzyme	Residue length	Start position	End position
Ect-A	DABA-Nγ-acetyltransferase	160	38792	39271
Ect-B	DABA transaminase	422	37423	38688
Ect-C	Ectoine synthase catalyses	139	36904	37320
EcT-D	Ectoine hydroxylase	304	35829	36740

**Table 4:**
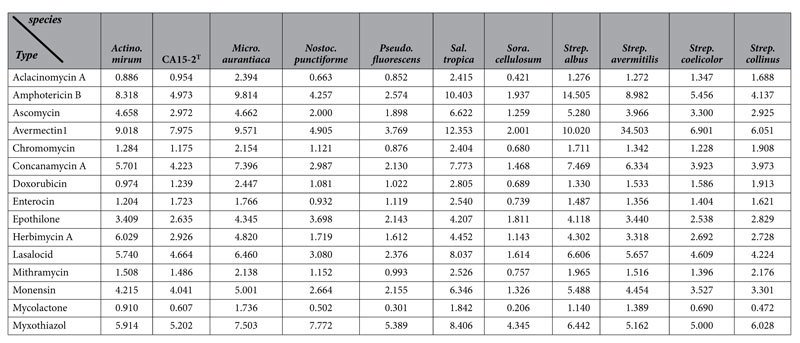
Q values of ten antibiotic-producing bacteria as well as *A. lopnorensis* CA15-2^T^ based on the blastp alignment against 15 gene clusters involved in antibiotic synthesis.

**Table 5:**
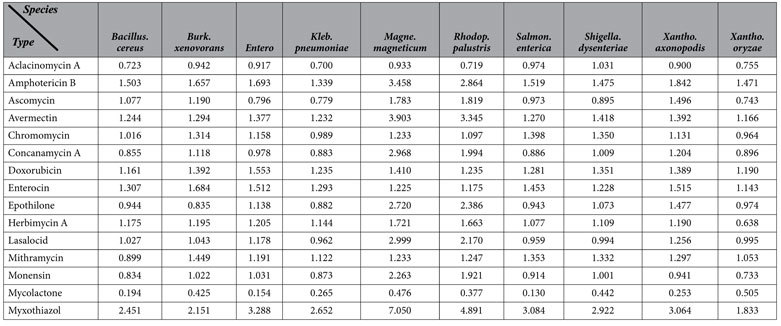
Q values of ten non-antibiotic-producing bacteria based on blastp alignment against 15 gene clusters involved in antibiotic synthesis.

**Table 6:**
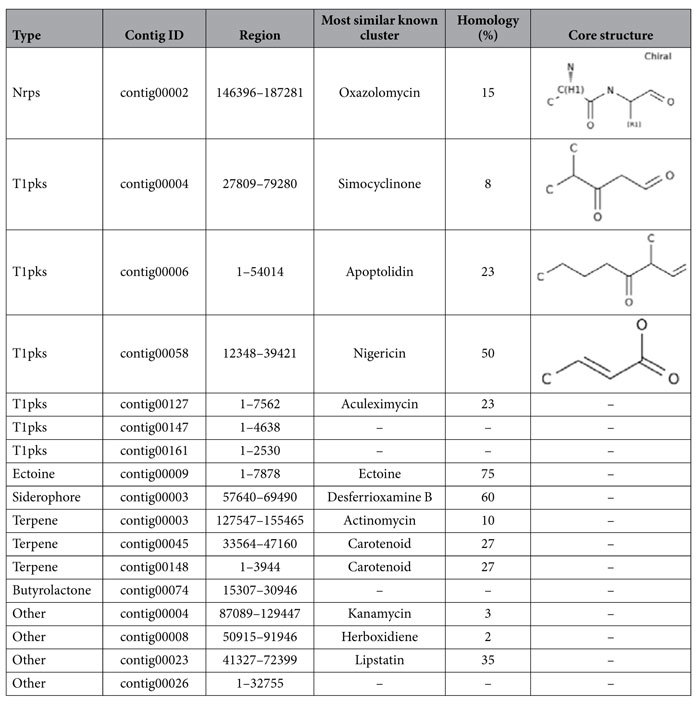
Overview of 17 secondary metabolic biosynthesis gene clusters of *A. lopnorensis* CA15-2^T^ detected by anti SMASH2.0 server.
