# Supplementary material for: Genome-guided Investigation of Antibiotic Substances produced by Allosalinactinospora lopnorensis CA15-2T from Lop Nor region, China
Source: Sci Rep. 2016 Feb 11;6:20667. doi: 10.1038/srep20667 (PMC4749953; doi:10.1038/srep20667)
Supplement: Supplementary Information [file srep20667-s1.doc]

**Supplementary Information**

Genome-guided Investigation of Antibiotic Substances produced by *Allosalinactinospora lopnorensis* CA15-2^T^ from Lop Nor region, China

Chen Huang^1†^, Ross Ka-KitLeung^2†^, Min Guo^1†^, Li Tuo^3^, Lin Guo^3^,YEW Wing Wai^4^, Inchio Lou^5^, Chenghang Sun^3*^ & Simon MY Lee^1*^

^1^State Key Laboratory of Quality Research in Chinese Medicine and Institute of Chinese Medical Sciences, University of Macau, Macao, China, ^2^Stanley HoCentre for Emerging Infectious Diseases, The Chinese University of Hong Kong, Shatin, New Territories, Hong Kong SAR, China, ^3^Department of Microbial Chemistry, Institute of Medicinal Biotechnology, Chinese Academy of Medical Sciences and Peking Union Medical College, Beijing 100050, China, ^4^Department of Microbiology, Chinese University of Hong Kong, ^5^Faculty of Science and Technology, Department of Civil and Environmental Engineering, University of Macau, Macao, China

†These authors contributed equally to this work

Correspondence and requests for materials should be addressed to S.M.L (simonlee@umac.mo) & C.H.S (sunchenghang@imb.pumc.edu.cn, or, chenghangsun@hotmail.com)

**Supplementary Figures**

**Figure S1. Representative growth image of** ***A. lopnorensis* CA15-2^T^ observed on R2A basal medium without NaCl (A) and with 5% NaCl (B).**


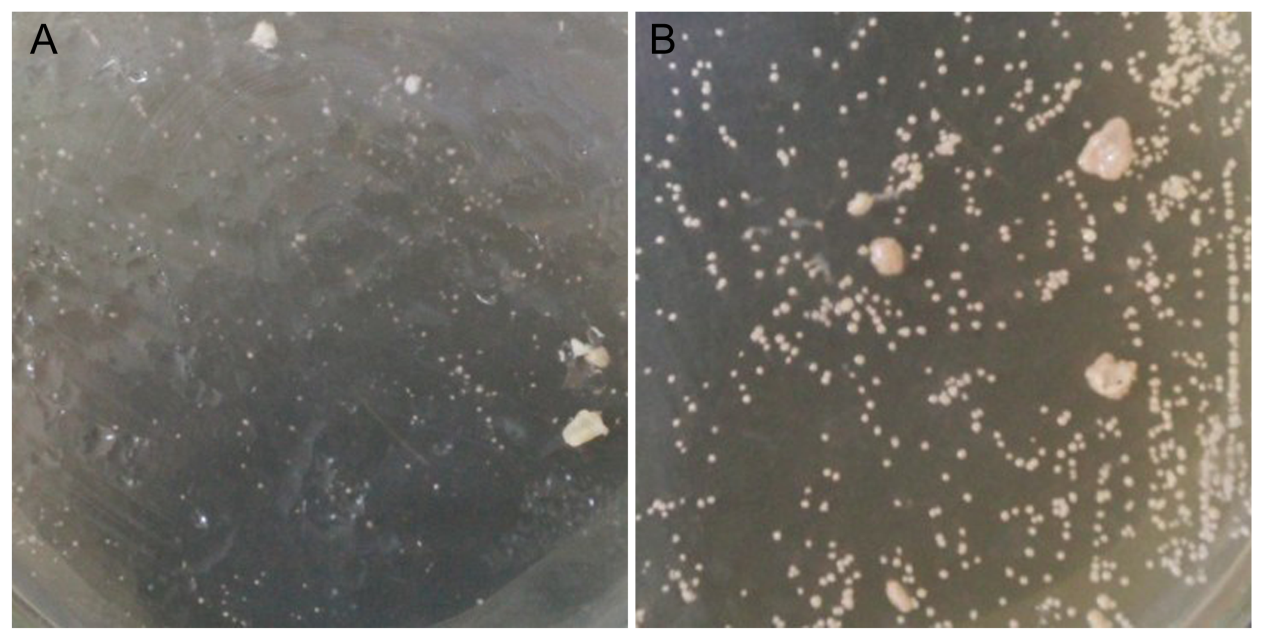


**Figure S2. The length distribution of 5549 predicted protein-coding genes of strain *A. lopnorensis* CA15-2^T^. Gene length is expressed as base pairs (bp) and the frequency as the absolute number of genes.**


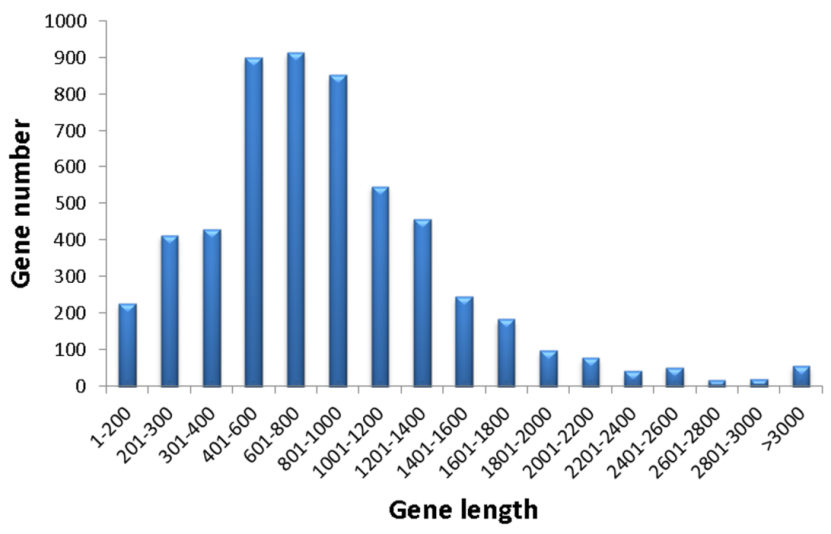


**Figure S3 eggNOG function classification of 5549 protein-coding sequences.**


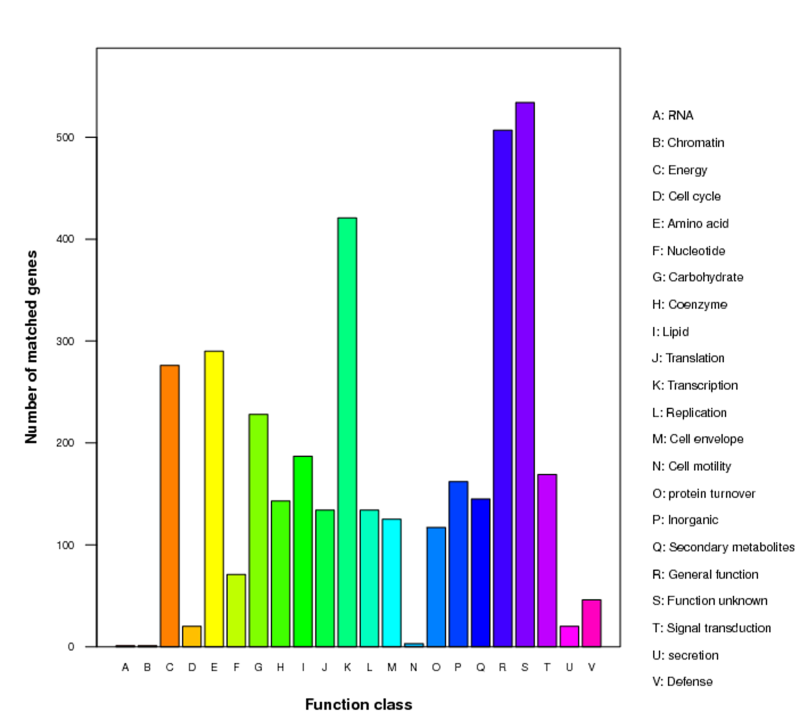


**Figure S4 Comparison of 16s rRNA sequences from strain *A. lopnorensis* CA15-2^T^ with other orthologous.** Phylogenetic tree based on maximum likelihood method of a muti-alignment of ten housekeeping genes, respectively. The number of boostrap replication was set to 500.


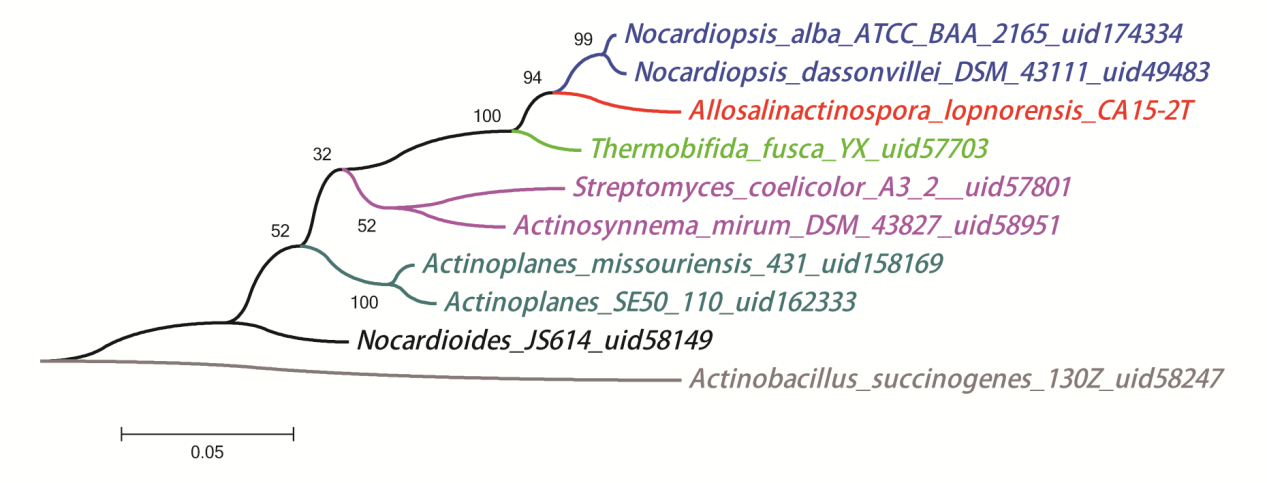


**Figure S5 A scores plot showing the relationship between antibiotics-producing group and non-antibiotics-producing group in terms of Principal Component 1 (x-axis) vs Principal Component 2 (y-axis) from Rotated Component Matrix.**


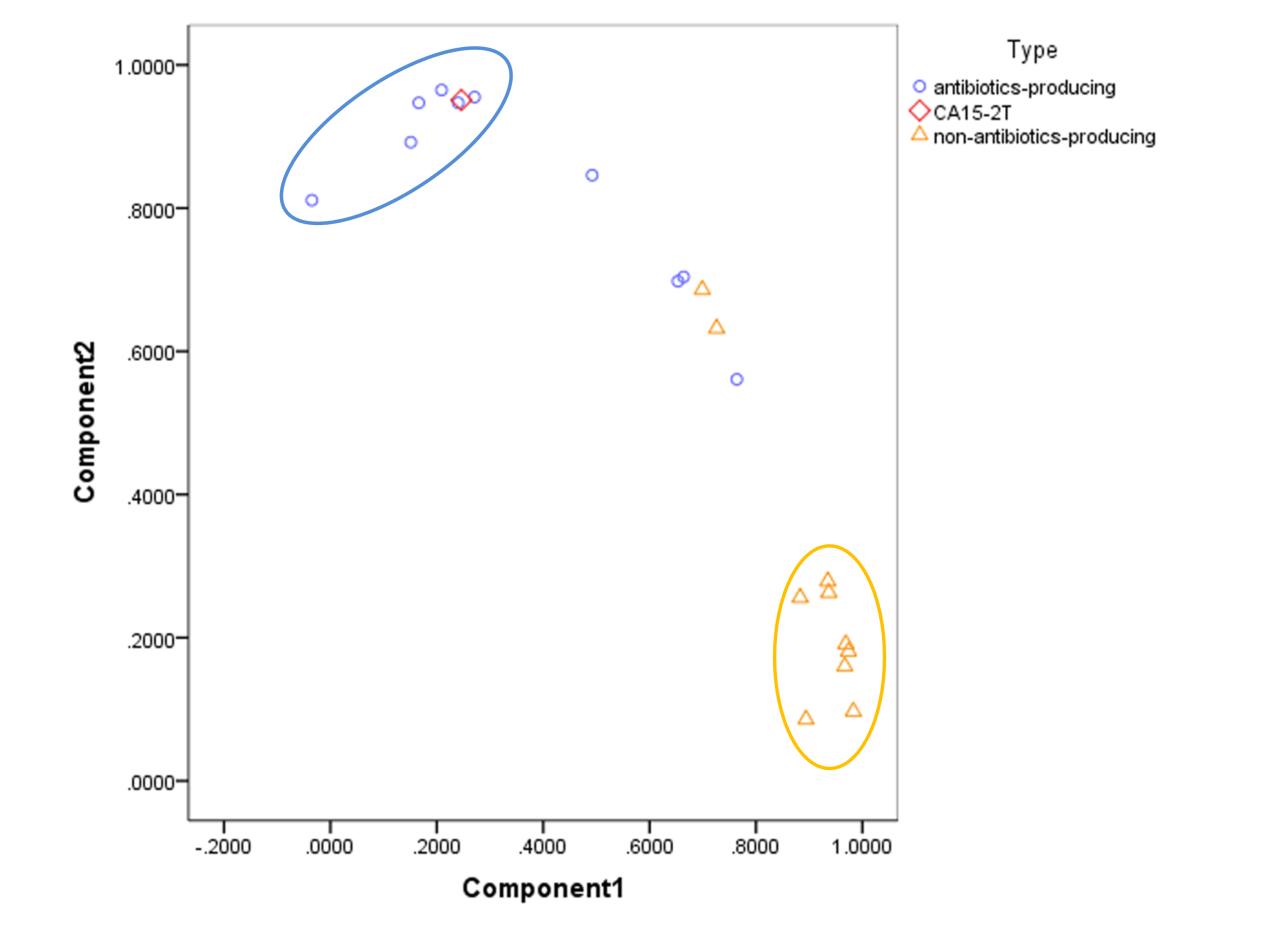


**Figure S6 Phylogenetic trees constructed based on 16s rRNA (A) and ten housekeeping gene (B) sequences among ten antibiotics-producing species (red color) and non-antibiotics-producing species (green color).**


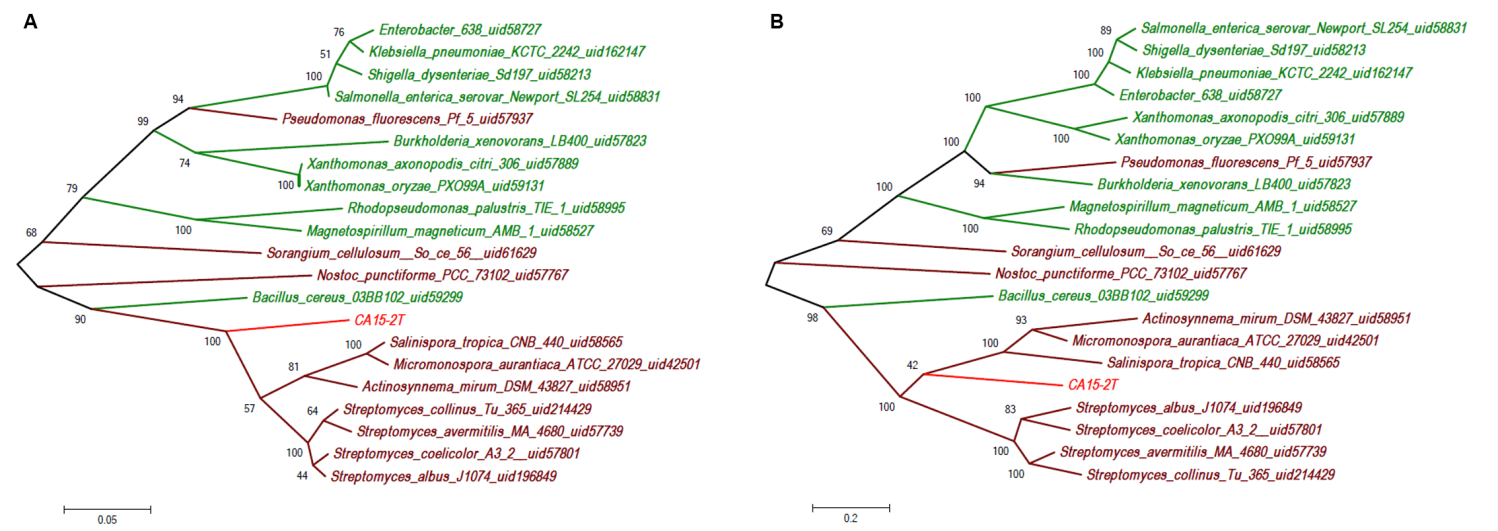


**Figure S7 Hierarchical cluster analyses of 15 antibiotics among twenty-one species.**


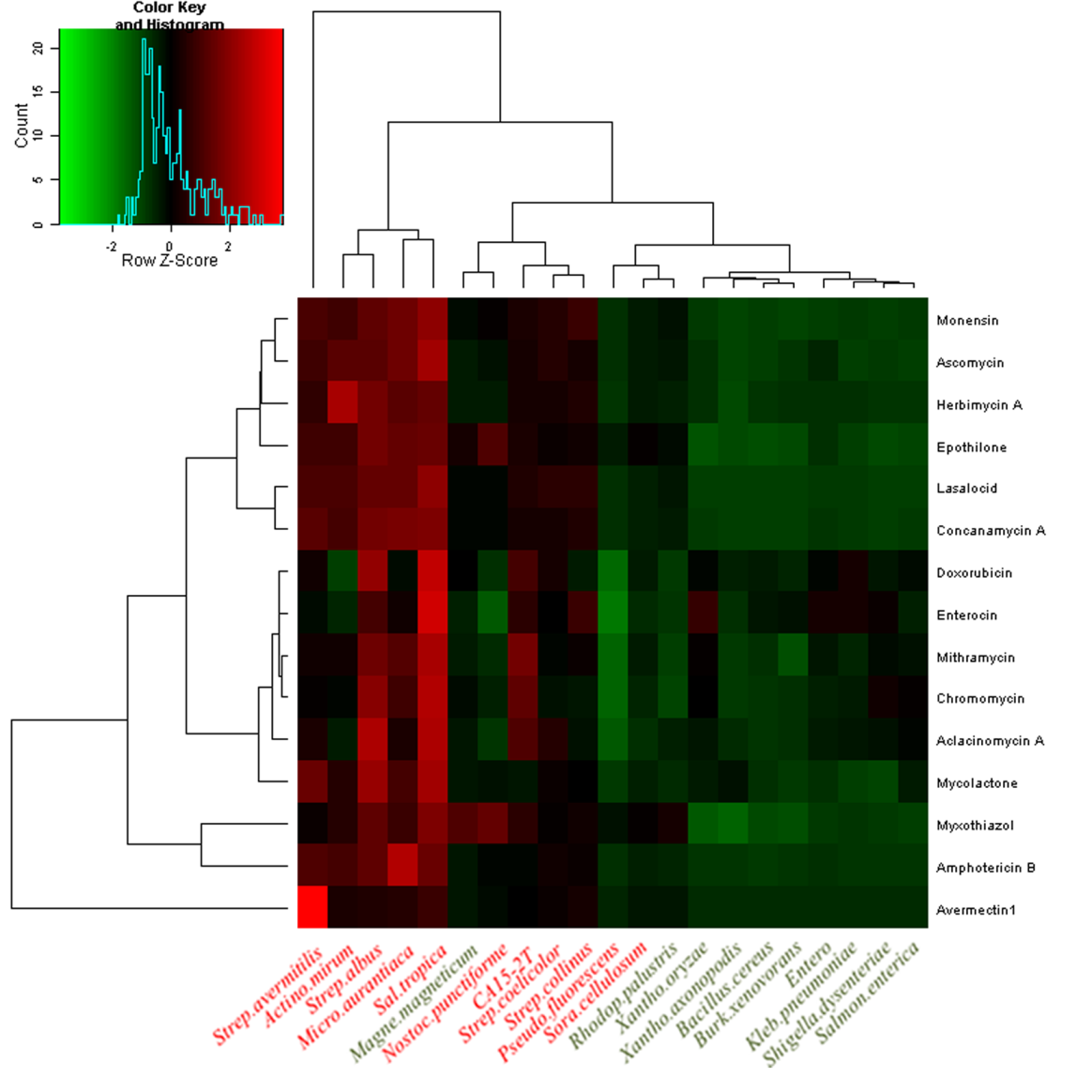


**Figure S8 Multiple alignments of the deduced amino acid sequences of 6-deoxyerythronolide-B synthase of simocyclinone cluster from *A. lopnorensis* CA15-2^T^ with known sequence from *Nocardiopsis dassonvillei* subsp. *dassonvillei* DSM 43111.** The identical and conserved amino acid residues are highlighted in black and gray, respectively. Dots represent gaps.

**
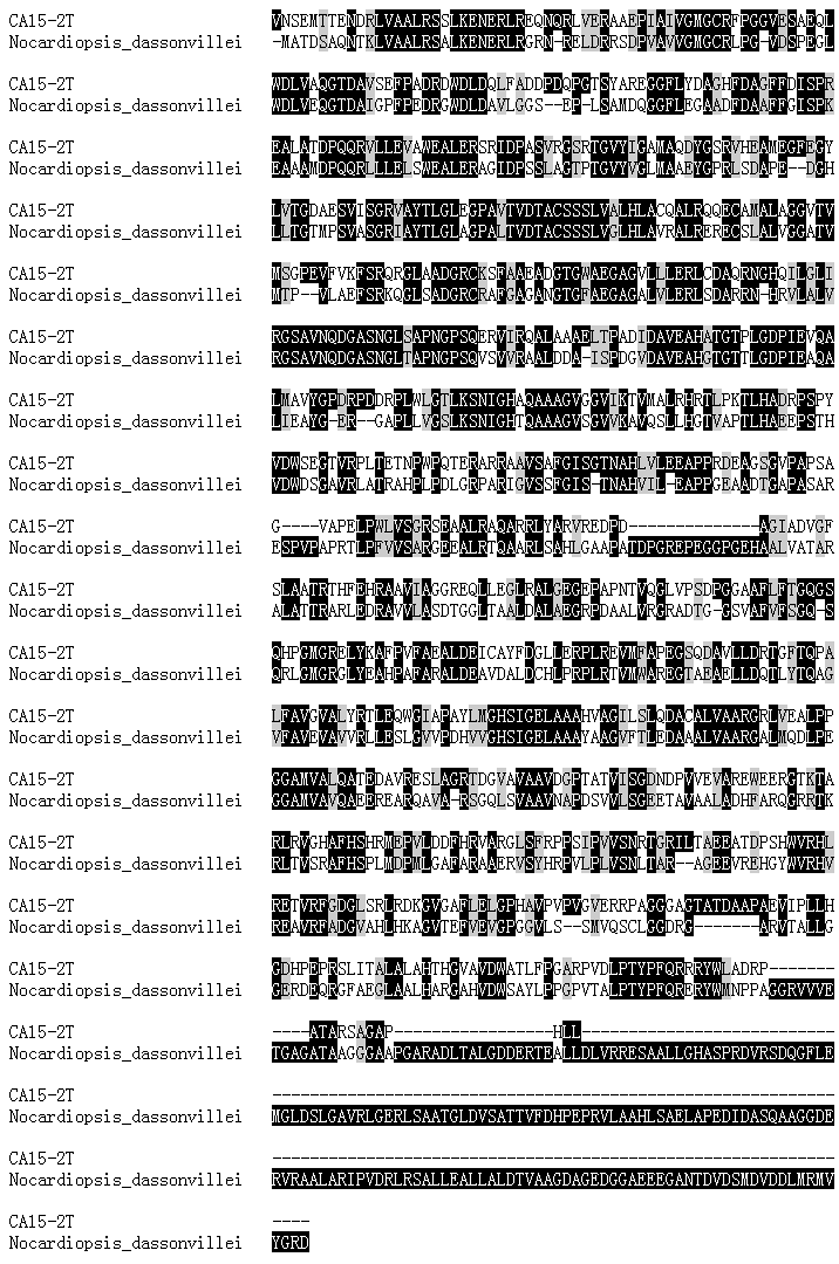
**

**Figure S9 Multiple alignments of the deduced amino acid sequences of acyl transferase from *A. lopnorensis* CA15-2^T^ with known sequence from *Nocardiopsis dassonvillei* subsp. *dassonvillei* DSM 43111.** The identical and conserved amino acid residues are highlighted in black and gray, respectively. Dots represent gaps.


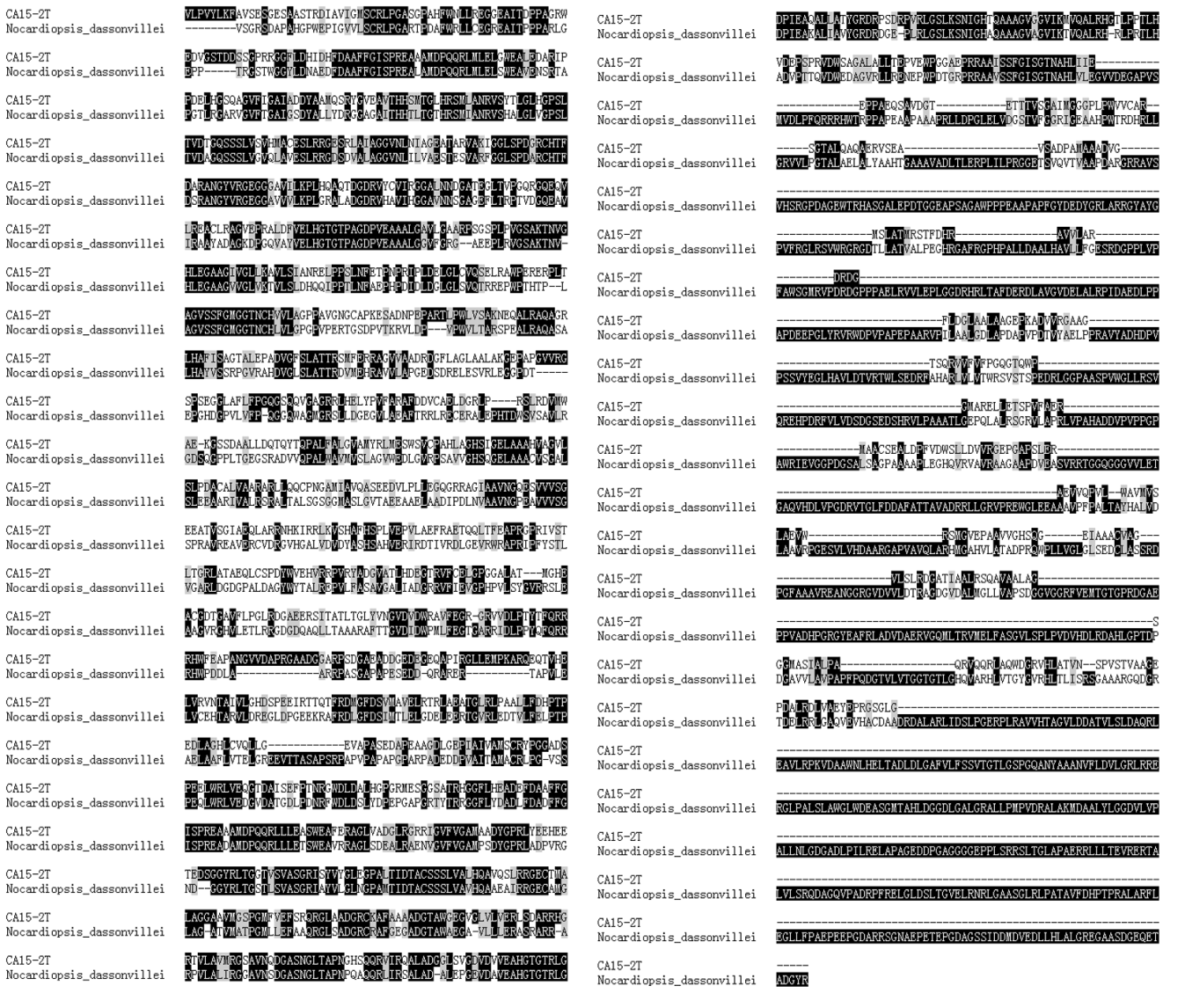


**Figure S10 Multiple alignments of the deduced amino acid sequences of Beta-ketoacyl synthase of nigericin cluster from *A. lopnorensis* CA15-2^T^ with known sequence from *Ktedonobacter racemifer* DSM 44963.** The identical and conserved amino acid residues are highlighted in black and gray, respectively. Dots represent gaps.


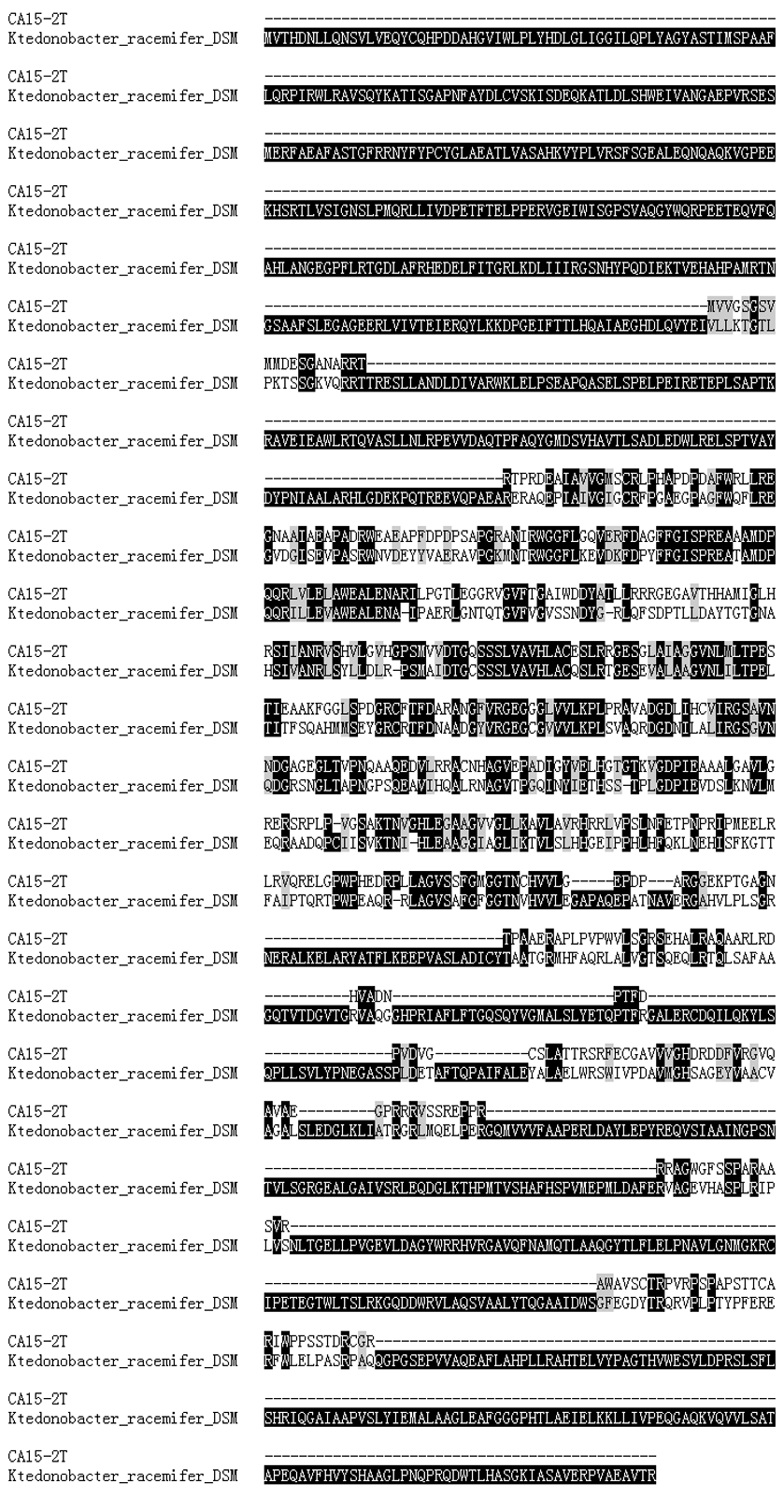


**Figure S11 Metabolic pathway of the compatible solute ectoine of *A. lopnorensis* CA15-2^T^.**


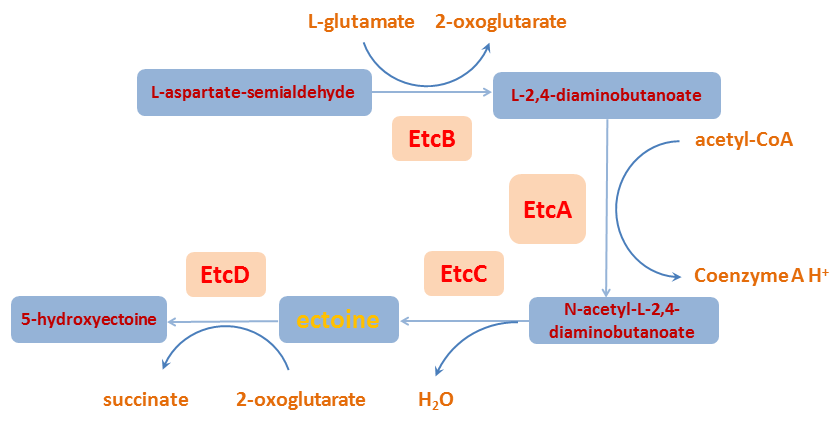


**Figure S12** **Putative Linear azol(in)e-containing peptide (LAP).** **A** putative LAP Gene cluster of *A. lopnorensis* CA15-2^T^; diverse colored arrows represent the gene of gene clusters within the genome sequence of *A. lopnorensis* CA15-2^T^. **B** Comparison of precursor peptides of streptolysin S (SagA), clostridiolysin S (ClosA) and plantazolicin (PlnA) with putative precursor peptides of *B. Hyodysenteriae* and *A. lopnorensis* CA15-2^T^ (highlighted in red); Cleavage site of leader and core peptides were connected with ‘—‘.


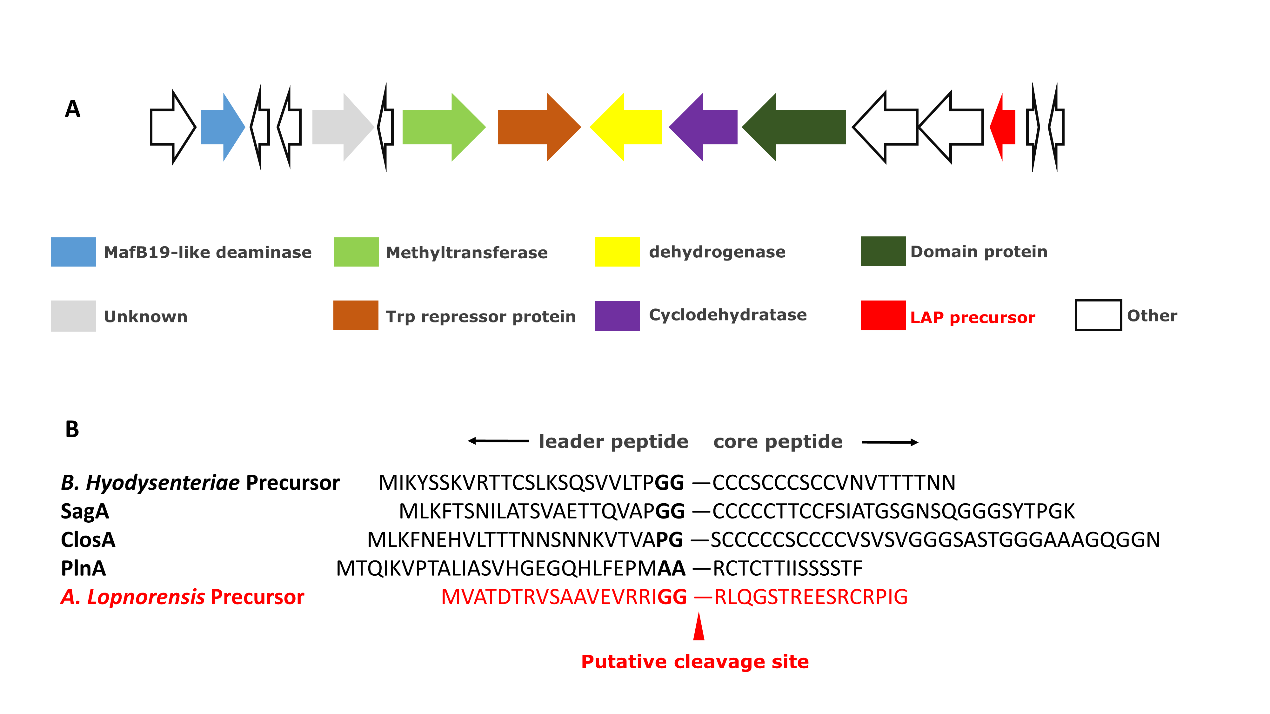


**Figure S13 Putative lasso peptide.** **A** Putative lasso Gene cluster of *A. lopnorensis* CA15-2^T^; diverse colored arrows represent the gene of gene clusters within the genome sequence of *A. lopnorensis* CA15-2^T^. **B** Sequences of lasso peptides of three main classes displaying the residues involved in the macrolactam in green; Hypothetical mecrolactam ring in putative lasso precursor peptide of *A. lopnorensis* CA15-2^T^ was shown. **C** Hypothetical formation processes of putative lasso peptide of *A. lopnorensis* CA15-2^T^ were displayed; Firstly, cleavage of putative lasso precursor peptide by putative ATP-dependent cysteine protease (brown arrow in Figure S13-A); Secondly, recognition peptide was cleaved at putative cleavage site according the length of existing lasso peptide; Finally, isopeptide bond (green) formation catalyzed by Asparagine synthase (purple arrow in Figure S13-A) between the N-terminal amino acid glycine (red) and Aspartic acid (red) leads to the formation of macrolactam ring in *A. lopnorensis* CA15-2^T^.


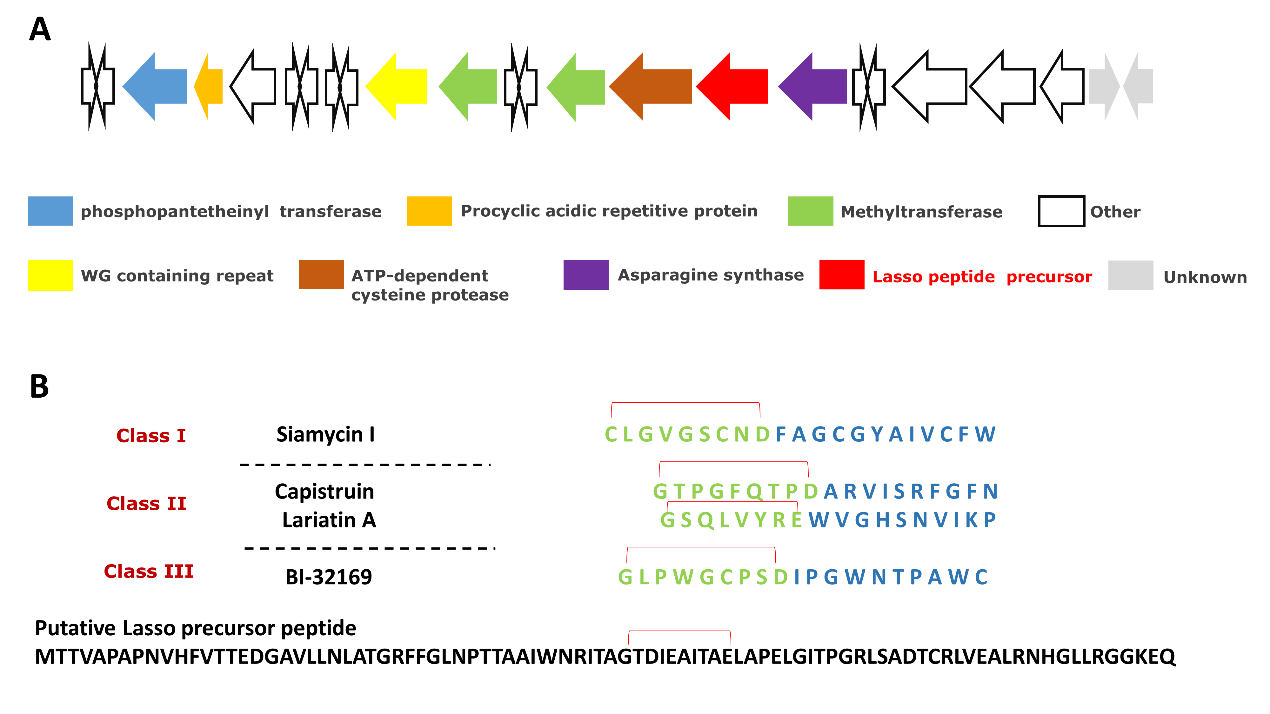

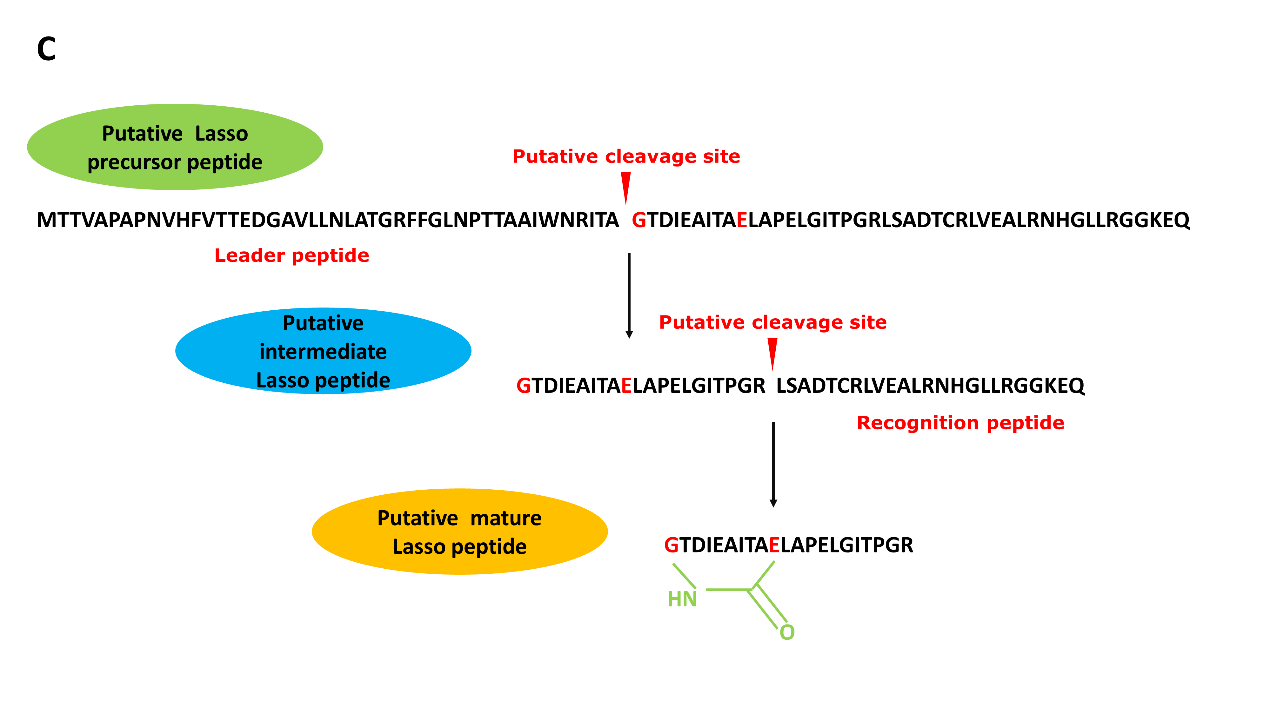


**Supplementary Tables**

**Table S1 Hit number of genes/PKS genes of ten non-antibiotic producing bacteria compared with 15 gene clusters of antibiotic synthesis based on blastp alignment.**

| **Antibiotics Name** | **Gene number/PKS gene number** | ***Bacillus.cereus*** | ***Burk.xenovorans*** | ***Entero*** | ***Kleb.pneumoniae*** | ***Magne.magneticum*** | ***Rhodop.palustris*** | ***Salmon.enterica*** | ***Shigella.dysenteriae*** | ***Xantho.axonopodis*** | ***Xantho.oryzae*** |
| --- | --- | --- | --- | --- | --- | --- | --- | --- | --- | --- | --- |
| Aclacinomycin A | 15/4 | 6/2 | 7/2 | 6/2 | 5/2 | 7/2 | 6/2 | 7/2 | 7/2 | 6/2 | 6/2 |
| Amphotericin B | 17/6 | 13/6 | 12/6 | 10/6 | 10/6 | 10/6 | 12/6 | 10/6 | 10/6 | 13/6 | 11/6 |
| Ascomycin | 22/3 | 11/3 | 12/3 | 7/3 | 9/3 | 12/3 | 13/3 | 9/3 | 8/3 | 12/3 | 9/3 |
| Avermectin | 6/4 | 5/4 | 4/4 | 4/4 | 4/4 | 4/4 | 5/4 | 4/4 | 4/4 | 4/4 | 4/4 |
| Chromomycin | 36/3 | 19/2 | 18/2 | 17/2 | 15/2 | 18/2 | 19/2 | 20/2 | 19/2 | 16/2 | 16/2 |
| Concanamycin A | 28/6 | 14/6 | 15/6 | 13/6 | 13/6 | 17/6 | 14/6 | 13/6 | 13/6 | 15/6 | 13/6 |
| Doxorubicin | 9/7 | 6/5 | 6/5 | 6/5 | 6/5 | 6/5 | 6/5 | 6/5 | 6/5 | 6/5 | 6/5 |
| Enterocin | 20/3 | 13/2 | 13/2 | 11/2 | 12/2 | 9/2 | 11/2 | 12/2 | 10/2 | 12/2 | 10/2 |
| Epothilone | 19/5 | 10/5 | 7/5 | 8/5 | 7/5 | 8/5 | 9/5 | 7/5 | 8/5 | 10/5 | 9/5 |
| Herbimycin A | 30/3 | 17/3 | 16/3 | 15/3 | 17/3 | 13/3 | 18/3 | 15/3 | 15/3 | 16/3 | 9/3 |
| Lasalocid | 24/7 | 13/7 | 11/7 | 13/7 | 12/7 | 13/7 | 12/7 | 12/7 | 12/7 | 13/7 | 13/7 |
| Mithramycin | 35/3 | 16/2 | 17/2 | 16/2 | 17/2 | 16/2 | 18/2 | 19/2 | 18/2 | 16/2 | 15/2 |
| Monensin | 34/10 | 19/9 | 18/9 | 16/9 | 16/9 | 15/9 | 19/9 | 16/9 | 16/9 | 16/9 | 15/9 |
| Mycolactone | 81/3 | 10/3 | 20/3 | 7/3 | 18/3 | 12/3 | 12/3 | 6/3 | 21/3 | 13/3 | 20/3 |
| Myxothiazol | 7/4 | 7/4 | 6/4 | 6/4 | 6/4 | 6/4 | 7/4 | 6/4 | 6/4 | 7/4 | 7/4 |

**Table S2 Hit number of genes/PKS genes of ten antibiotic producing bacteria as well as *A. lopnorensis* CA15-2^T^ compared with 15 gene clusters of antibiotic synthesis based on blastp alignment.**

| **Antibiotics Name** | **Gene number/PKS gene number** | ***Actin.miru*** | ***CA15-2T*** | ***Micro.aurantiac*** | ***Myco.ulceran*** | ***Nostoc.punctiform*** | ***Pseud.fluorescens*** | ***Soran.cellulosum*** | ***Strep.albus*** | ***Strep.avermitilis*** | ***Strep.coelicolor*** | ***Strep.collinus*** |
| --- | --- | --- | --- | --- | --- | --- | --- | --- | --- | --- | --- | --- |
| Aclacinomycin A | 15/4 | 8/2 | 7/2 | 15/4 | 9/2 | 6/2 | 8/2 | 6/2 | 10/3 | 13/3 | 12/4 | 14/4 |
| Amphotericin B | 17/6 | 15/6 | 12/6 | 13/6 | 12/6 | 11/6 | 10/6 | 12/6 | 16/6 | 15/6 | 15/6 | 14/6 |
| Ascomycin | 22/3 | 18/3 | 15/3 | 18/3 | 14/3 | 14/3 | 14/3 | 15/3 | 17/3 | 18/3 | 16/3 | 17/3 |
| Avermectin | 6/4 | 5/4 | 5/4 | 5/4 | 5/4 | 5/4 | 5/4 | 5/4 | 5/4 | 6/4 | 5/4 | 5/4 |
| Chromomycin | 36/3 | 24/2 | 19/2 | 33/3 | 20/2 | 22/2 | 17/2 | 18/2 | 28/2 | 27/3 | 28/3 | 31/3 |
| Concanamycin A | 28/6 | 25/6 | 20/6 | 24/6 | 19/6 | 21/6 | 17/6 | 21/6 | 20/6 | 22/6 | 21/6 | 24/6 |
| Doxorubicin | 9/7 | 6/5 | 7/6 | 9/7 | 6/5 | 6/5 | 6/5 | 6/5 | 7/6 | 8/7 | 8/7 | 8/7 |
| Enterocin | 20/3 | 15/2 | 16/2 | 17/3 | 13/2 | 10/2 | 14/2 | 13/2 | 15/2 | 16/2 | 16/3 | 18/3 |
| Epothilone | 19/5 | 10/5 | 9/5 | 11/5 | 8/5 | 11/5 | 8/5 | 13/5 | 9/5 | 9/5 | 10/5 | 10/5 |
| Herbimycin A | 30/3 | 24/3 | 22/3 | 23/3 | 20/3 | 21/3 | 19/3 | 19/3 | 21/3 | 24/3 | 26/3 | 25/3 |
| Lasalocid | 24/7 | 18/7 | 17/7 | 18/7 | 15/7 | 15/7 | 13/7 | 16/7 | 17/7 | 18/7 | 18/7 | 18/7 |
| Mithramycin | 35/3 | 28/2 | 23/2 | 32/3 | 20/2 | 21/2 | 17/2 | 19/2 | 28/2 | 28/3 | 25/3 | 32/3 |
| Monensin | 34/10 | 24/10 | 25/10 | 25/10 | 24/10 | 21/10 | 22/9 | 22/9 | 26/10 | 28/10 | 26/10 | 27/10 |
| Mycolactone | 81/3 | 12/3 | 17/3 | 21/3 | 33/3 | 9/3 | 8/3 | 10/3 | 10/3 | 28/3 | 14/3 | 9/3 |
| Myxothiazol | 7/4 | 6/4 | 7/4 | 7/4 | 6/4 | 7/4 | 6/4 | 7/4 | 6/4 | 7/4 | 6/4 | 7/4 |

**Table S3 Multivariate Tests^a^.**

| Effect | | Error df | Sig. |
| --- | --- | --- | --- |
| Gene Clutser Type | Pillai’s Trace | 5.000 | 0.001 |
|  | Wilks' Lambda | 5.000 | 0.001 |
|  | Hotelling's Trace | 5.000 | 0.001 |
|  | Roy's Largest Root | 5.000 | 0.001 |
| Gene Cluster Type * group | Pillai's Trace | 5.000 | 0.096 |
|  | Wilks' Lambda | 5.000 | 0.096 |
|  | Hotelling's Trace | 5.000 | 0.096 |
|  | Roy's Largest Root | 5.000 | 0.096 |

**a. Design: Intercept + group**

**Within Subjects Design: clustertype**

**Table S4 Tests of Between-Subjects Effects.**

| Source | Type III Sum of Squares | df | Mean Square | F | Sig. |
| --- | --- | --- | --- | --- | --- |
| Intercept | 1921.981 | 1 | 1921.981 | 104.516 | 0.000 |
| group | 396.791 | 1 | 396.791 | 21.577 | 0.000 |
| Error | 331.008 | 18 | 18.389 |  |  |

**Table S5 Basically information of 15 antibiotic compounds extracted from DOBISCUIT database (version: 2014-06-25).**

| **Compound name** | **PKS Type** | **organism** | **activity** |
| --- | --- | --- | --- |
| Aclacinomycin A | TypeII | Streptomyces galilaeus ATCC 31615 | Antitumor; Toxic |
| Amphotericin B | TypeI | Streptomyces nodosus ATCC 14899 | Antifungal |
| Ascomycin | PKS-NRPS | Streptomyces hygroscopicus subsp. ascomyceticus ATCC 14891 | Immunosuppressive; Antifungal |
| Avermectin | TypeI | Streptomyces avermitilis ATCC 31267 | Anthelmintic |
| Chromomycin | TypeII | Streptomyces griseus ATCC 13273 | Antibacterial; Antitumor; Antiviral |
| Concanamycin A | TypeI | Streptomyces neyagawaensis ATCC 27749 | Antifungal; Antiprotozoal; Antitumor; Antiviral |
| Doxorubicin | TypeII | Streptomyces peucetius subsp. caesius | Antitumor |
| Enterocin | TypeII | Streptomyces maritimus | Antibacterial |
| Epothilone | PKS-NRPS | Sorangium cellulosum So ce90 | Antitumor |
| Herbimycin A | TypeI | Streptomyces hygroscopicus AM 3672 | Antitumor |
| Lasalocid | TypeI | Streptomyces lasaliensis ATCC 35851 | Antibacterial; Ionophore |
| Mithramycin | TypeII | Streptomyces argillaceus ATCC 12956 | Antibacterial; Antitumor |
| Monensin | TypeI | Streptomyces cinnamonensis ATCC 15413 | Antibacterial; Ionophore; Antiprotozoal |
| Mycolactone | TypeI | Mycobacterium ulcerans Agy99 | Toxic; Immunosuppressive |
| Myxothiazol | PKS-NRPS | Stigmatella aurantiaca DW4/3-1 | Antifungal |

**Table S6 summary of Genome and gene amount of ten antibiotics-producing species and non-antibiotics-producing species.**

| Anti-species | Genome size | Gene amount | Non-anti species | Genome size | Gene amount | Feature |
| --- | --- | --- | --- | --- | --- | --- |
| *Actin.miru* | 8248144 | 6916 | *Bacillus.cereus* | 5269628 | 5398 | pathogenicity |
| *Micro.aurantiac* | 7025559 | 6222 | *Burk.xenovorans* | 4895836 | 4430 | pathogenicity |
| *Myco.ulceran* | 5631606 | 4160 | *Entero* | 4518712 | 4115 | enterobacterium |
| *Nostoc.punctiform* | 8234322 | 6086 | *Kleb.pneumoniae* | 5259571 | 4923 | pathogenicity |
| *Pseud.fluorescens* | 7074893 | 6108 | *Magne.magneticum* | 4967148 | 4561 | magnetotropism |
| *Soran.cellulosum* | 13033779 | 9380 | *Rhodop.palustris* | 5744041 | 5246 | tolerance |
| *Strep.albus* | 6841649 | 5832 | *Salmon.enterica* | 4827641 | 4605 | pathogenicity |
| *Strep.avermitilis* | 9025608 | 7580 | *Shigella.dysenteriae* | 4369232 | 4268 | pathogenicity |
| *Strep.coelicolor* | 8667507 | 7768 | *Xantho.axonopodis* | 5175554 | 4312 | pathogenicity |
| *Strep.collinus* | 8272925 | 7005 | *Xantho.oryzae* | 5240075 | 4988 | pathogenicity |
